# Geochemistry of some fluoride and nitrate enriched water resources from the Oriental Basin: a prospective health risk hotspot from eastern-central Mexico

**DOI:** 10.1007/s10653-025-02421-z

**Published:** 2025-03-13

**Authors:** Priyadarsi D. Roy, Oscar Agesandro García-Arriola, Sekar Selvam, Irma Gabriela Vargas-Martínez, José Luis Sánchez-Zavala

**Affiliations:** 1 Instituto de Geología, Universidad Nacional Autónoma de México, Del. Coyoacán, CP 04510 Mexico City, Mexico; 2 Posgrado en Ciencias del Mar y Limnología, Universidad Nacional Autónoma de México, Del. Coyoacán, CP 04510 Mexico City, Mexico; 3 Department of Geology, V.O. Chidambaram College, Tuticorin, Tamil Nadu 628008 India; 4 Carrera de Ingeniería Geológica, Facultad de Ingeniería, Universidad Nacional Autónoma de México, Del. Coyoacán, CP 04510 Mexico City, Mexico

**Keywords:** Ion chemistry, Drinking water quality index, Libres-Oriental aquifer, Totolcingo Lake, Non-carcinogenic risk, Mexico

## Abstract

In attention to the Sustainable Development Goal 6, the quality evaluation of water resources in Mexico is limited compared to other regions. This study provided new data from Oriental Basin, an important socio-economic region with up to 20% population growth over the last decade by assessing groundwater from the Libres-Oriental aquifer (Ca–Mg–HCO_3_ facies; F^−^: 2.5–9.9 mg/L; NO_3_^−^: up to 75.3 mg/L) and water from the Totolcingo Lake (Na–Cl facies; F^−^: 12.7–13.2 mg/L; NO_3_^−^: < 0.75 mg/L). Fluoride content grouped about 80% groundwater samples as promotor of dental and skeletal fluorosis. Nitrate Pollution Index suggested moderate pollution in 20% and very significant pollution in 10% groundwater samples. Possible exposure of older adults and elderly pregnant women to fluorosis from all the groundwater samples (Hazard Quotient > 1) from the Oriental Basin and 55% of them might also be causing fluorosis in infants suggest a potential health risk hotspot in the eastern-central Mexico. Even though all the groundwater samples did not contain enough NO_3_^−^ to cause methemoglobinemia, their boiling for drinking could enhance nitrate content beyond the WHO limit. Thus, the mitigation techniques might diminish the health risks in consuming population.

**Supplementary Information:**

The online version contains supplementary material available at 10.1007/s10653-025-02421-z.

## Introduction

About 2.2 billion global population, mostly from the developing countries, presently lacks access to the safe drinking water due to recurrent droughts, and because almost 80% of the untreated wastewater is allowed to flow back into the ecosystem (United Nations, 2023). Thus, the Sustainable Development Goal 6 (SDG 6), as part of the 2030 agenda, provides the blueprint for ensuring availability of safe water and sustainable management of the water resources. However, the usual pollutants like fluoride (F^−^) and nitrate (NO_3_^−^), both from the geogenic and anthropogenic sources, have been consistently affecting the water quality across the globe. The World Health Organization (WHO) has recommended F^−^ below 1.5 mg/L and NO_3_^−^ below 50 mg/L for the safe consumption as the drinking water with both contaminants above the recommended values could cause dental/skeletal fluorosis as well as birth defect, blue-baby syndrome (methemoglobinemia) and lower IQ levels (Abascal et al., 2022; Alarcón-Herrera et al., 2020; Kimambo et al., 2019; Shaji et al., 2024).

In a state-of-the-art global fluoride hazard map with over 400,000 measurements in groundwater, Podgorski and Berg (2022) observed health risk hotspots in the central Australia, western North America, and eastern Brazil and that up to 59% of total population in Asia and up to 46% of total population in Africa consume drinking water with high fluoride. Around 0.3% population of North America are exposed to groundwater with F^−^ > 1.5 mg/L and around 20 million people, including 6.5 million children in Mexico, are exposed to F-enriched water (Alarcón-Herrera et al., 2020; Podgorski & Berg, 2022). About 3 million people of Mexico presently reside within 5 km of a fluoride enriched groundwater well (Alarcón-Herrera et al., 2020). It has led to dental and skeletal fluorosis and even osteosclerosis in the higher concentrations (F^−^ > 4 mg/L) (Kimambo et al., 2019; Ochoa-Rivero et al., 2023; Shaji et al., 2024; WHO, 2017). Some of the anthropogenic sources such as phosphatic fertilizer, coal combustion, aluminum smelting, and cement manufacture contribute fluoride to the water resources (Dey et al., 2012; Shaji et al., 2024). The principal mechanism for fluoride enrichment, however, is chemical weathering of the volcanic lithologies (rhyolite, ignimbrites, ash of variable compositions, and glasses), metamorphic rocks and limestone with fluoride-bearing minerals (Navarro et al., 2017; Reyes-Gómez et al., 2017; Roy et al., 2022; Shaji et al., 2024). Under the conditions of prolonged droughts and alkaline environments, the F-bearing minerals release F^−^ into Na^+^ enriched water resources (Alvarez & Carol, 2019; Crundwell, 2017; Shaji et al., 2024).

In contrast, the contribution of nitrate from geogenic sources is negligible as it is principally derived from the anthropogenic sources, such as nitrogen-rich fertilizers used in agriculture, contamination from poorly treated domestic and industrial wastewater, leaching from the livestock manure and landfill sites into surface and groundwater, as well as the inadequate sanitation management in developing countries (Abascal et al., 2022; Kapembo et al., 2016; Tokazhanov et al., 2020). About 30 regions in Africa, 20 in Asia, and 9 in Europe are in a critical situation from the nitrate contamination (Abascal et al., 2022). Exposure to high nitrate (> 50 mg/L) could cause methemoglobinemia, thyroid or cancer, hypertension, diabetes, spontaneous abortion, respiratory tract infection and change in the immune system (Fewtrell, 2004; Gupta et al., 2000; Martínez et al., 2017; Tokazhanov et al., 2020).

In Mexico, the surface water resources provide 60.6% and groundwater contributes 39.4% of the total consumptions, and both have increased by 23% and 42%, respectively, over the last two decades due to the swelling demands from agriculture, public supply and industry (CONAGUA, 2022). Lake Chapala with a storage capacity of 8126 hm^3^ (cubic hectometers) presently stores less than 4871 hm^3^ due to the climate change and only 378 aquifers out of the total 653 aquifers in Mexico are in surplus conditions due to persistent droughts over the last two decades (CONAGUA, 2022). The water quality studies in Mexican rivers and lakes are scarce. For example, the study of Gradilla-Hernández et al. (2020) reported that the Lake Cajititlán of western Mexico received excess fertilizer runoff from the nearby agricultural camps and approximately 2.3 hm^3^/yr of poorly treated wastewater. Roy et al. (2023) observed up to 4.3 mg/L of fluoride in water samples from the Lake Coatetelco of central-southern Mexico. In comparison, the groundwater resources are better monitored and the data from wells in several semi-arid and arid states (annual precipitation < 700 mm) in central and northern Mexico (San Luis Potosi, Zacatecas, Chihuahua, and Sonora) showed up to 29.6 mg/L of F^−^, i.e., about 20-fold higher than the WHO recommendation (Alarcón-Herrera et al., 2020). About 65% groundwater samples from the semi-arid Chihuahua City contained F^−^ above the WHO guideline (Ochoa-Rivero et al., 2023) and groundwater chemistry from the Independent Basin of central Mexico showed up to 15.5 mg/L of F^−^ (LaFayette et al., 2020). Roy et al. (2021) observed F^−^ > 1.5 mg/L in 20–25% wells in the El Potosi and Sandia basins of northeast Mexico.

Similarly, the nitrate content in wells of Linares (average: 80 mg/L) was sourced mainly from the urban, agricultural, and livestock activities (de León‑Gómez et al., 2020). More than 25% samples of the Mezquital and Toluca Valley and 32% of groundwater wells in the Comarca Lagunera had NO_3_^−^ above 50 mg/L (de Oca et al., 2019; Esteller et al., 2017; Torres-Martínez et al., 2021). However, the water quality monitoring needs to expand and cover all the surface water bodies as well as aquifers for more effective mitigation and management strategies. Methemoglobinemia risk from the water consumption is minimal compared to the more prevalent fluorosis risk in Mexico (Calleros et al., 2012; Betancourt-Lineraes et al., 2013; ENCD, 2018). The National Dental Caries Survey of 2001 observed an overall fluorosis prevalence of 27.9% in 27,000 students of 12 and 15 years old with the highest value registered in Durango state (87.5%) and prevalence of more than 50% in the arid states of Baja California, San Luis Potosi, Zacatecas and Aguascalientes (Betancourt-Lineraes et al., 2013). The survey of 2011–2014, however, revealed a changed scenario about dental fluorosis with > 50% prevalence in more humid states of Yucatan, Veracruz, Tamaulipas, Oaxaca and Chiapas (ENCD, 2018). The survey of 2001 indicated an overall caries prevalence of 32.9% in the Puebla state of eastern-central Mexico, and its subsequent increase to > 50% in the survey of 2011–2014, i.e., after a decade (ENCD, 2018).

This study attempts to evaluate the water quality in terms of fluoride and nitrate enrichments from the deficit Libres-Oriental aquifer and the Totolcingo Lake in Oriental Basin of the Puebla state, a region of socio-economic importance, for their suitability as drinking water resources and estimate the possible non-carcinogenic health risks for infants, children, older adults, and vulnerable pregnant women from the hazard quotient (HQ) values and by estimating the nitrate pollution index as well as the drinking water quality index (DWQI). The physicochemical characteristics were related with the internationally accepted WHO guidelines to contribute to the water quality database comparable in a global-scale.

## Study area

**Fig. 1:**
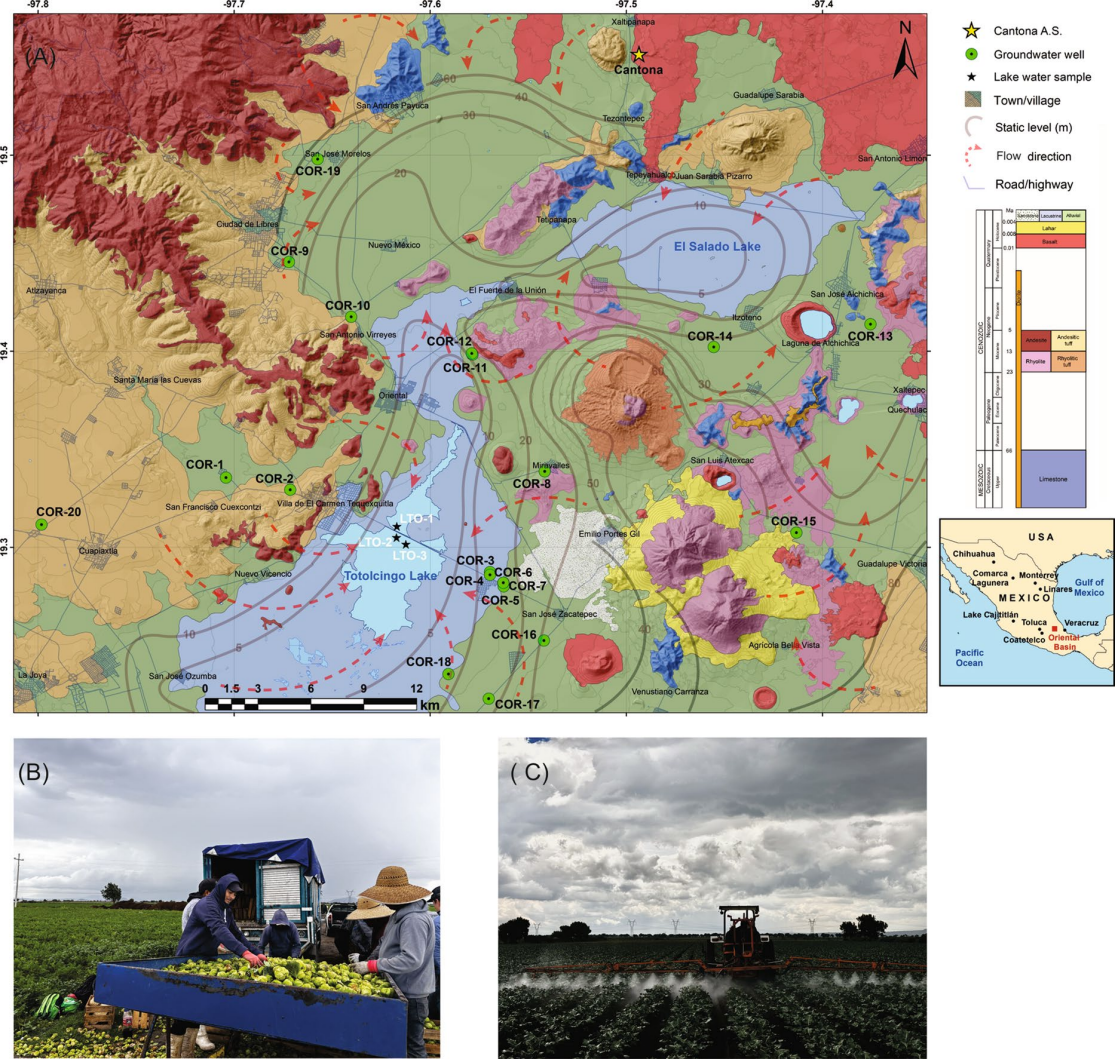
**A** Map showing location of groundwater wells and samples from Totolcingo Lake in the Oriental Basin of eastern-central Mexico and some other discussed sites. Photographs showing harvesting of tomatoes along with poultry manure heaps in the background as fertilizer (**B**) and application of synthetic fertilizer in an agriculture field (**C**)

The Oriental Basin (Puebla state) of Mexico is a volcano-sedimentary basin and it is limited by the Trans Mexican Volcanic Belt in the east, mountains of the Sierra Madre Oriental and coastal plains of the Gulf of Mexico in the north and east, and mountains of the Sierra Madre del Sur in the south (Fig. 1). It has mostly semi-arid temperate climate (CONAGUA, 2023). Data of 1981–2010 from the meteorological stations of the National Meteorological Service of Mexico at Villa de El Carmen (west), San Luis Atexcac (east) and Tepeyahualco (north) registered average annual precipitation between 454 and 500 mm from 56 to 78 days/year, with > 75% of them occurring in the summer and autumn months (May-and-September), and more than threefold higher annual evaporation (1580–1889 mm; source: https://smn.conagua.gob.mx/es/climatologia/informacion-climatologica/informacion-estadistica-climatologica).

Geology of this basin is considered from the Mexican Geological Service maps, and it shows outcrops of Mesozoic to Cenozoic sedimentary rocks, such as limestone, sandstone and alluvial-lacustrine sediments, as well volcanic lithologies, such as rhyolite, andesite, basalt, and diorite in the basin (Fig. 1A, Servicio Geologico Mexicano, 2011a, 2011b, 2012). Limestone (Albian-Cenomanian) is the oldest sedimentary unit with outcrops in northern, northeastern, south-central and southeastern portions of the basin. It is stratified with bands of black chert and shale. The Cenozoic lithologies are represented by andesite, andesitic tuff, rhyolite and rhyolite tuff from the magmatic events of the Trans-Mexican Volcanic Belt and an intrusion of diorite. The Quaternary deposits are made up spills and basaltic breccia and products of the monogenetic cones and the Los Humeros volcanic field as well as the unconsolidated lacustrine and alluvial deposits (CONAGUA, 2023). The most outstanding structural features correspond to a system of folds and NW–SE thrusts at the northwestern basin margin (CONAGUA, 2023).

The Libres-Oriental aquifer and the Totolcingo Lake are two important hydrological resources in the endorheic Oriental Basin with centrifugal sub-dendritic and rectangular drainages with its recharge areas on the volcanic and sedimentary mountain ranges. The surface runoff is intermittent and scarce with seasonal streams from the northwest bringing runoff to the lake. This aquifer is unconfined to semiconfined, heterogenous, and anisotropic with a surface area of ca. 3995 km^2^ and it belongs to the administrative hydrological region Balsas (region IV, Mexico). It is comprised of unconsolidated alluvial and lacustrine deposits as well as volcanic rocks in the upper part and limestone in the lower part (CONAGUA, 2023). Its static level varies between 5 and 160 m, and the over-extraction of groundwater in last two decades has reduced the static level at 0.3–0.6 m/year in different parts of the basin. Hydraulic transmissivity of this aquifer is variable with 0.01–13.70 × 10^–3^ m^2^/s. Higher annual extraction (157.9 hm^3^) compared to the annual recharge (137.4 hm^3^) characterizes its deficit nature (CONAGUA, 2023). A significant 16% of the extraction is used for rural–urban consumptions through the public supply and about 81.4% of the extracted groundwater is presently used for agricultural needs (CONAGUA, 2023). The Totolcingo Lake is still perennial but presently reduced to a shallow water body (< 1 m) with a coverage of about 50 km^2^ during the high stand. It has a maximum length of ca.12.5 km and maximum width of ca. 5 km in a NE-SW orientation in central part of the basin (Fig. 1A). The static level remains below 5 m under the lake, suggesting the connection between lake and aquifer during the wet seasons. Both the groundwater as well as surface water is presently used by 117,463 inhabitants residing in five surrounding municipalities and all these municipalities have witnessed between 11 and 20% of population growth over the last decade (source: www.datamexico.org).

## Sampling and analysis

Most of the precipitation (> 75%) in Oriental Basin occurs between May and September, and the month of April is characterized by above average temperature (15.5–16.4 °C; average annual 13.9–14.5 °C) and minimal rainfall (29–36 mm; average annual 454–500 mm). Water samples were collected in PTFE (Polytetrafluoroethylene) bottles from 20 groundwater wells (COR-1 to COR-20) with water depths between 5 and 60 m in the Libres-Oriental aquifer after the pump purging and 3 water samples from the Totolcingo Lake (LTO-1 to LTO-3) in April 2022 (prior to the rainy summer-autumn) as the objective was to evaluate their physicochemical characteristics in an enriched state and assess the health risks through F^−^ and NO_3_^−^ consumptions (Fig. 1A). All the groundwater wells sampled in this study are used for irrigation and consumption. Electrical conductivity (EC), total dissolved solids (TDS) and Hydrogen ion concentration (pH) were measured in the field with a portable instrument (Hanna HI 98130), and the samples were subsequently transported under 4 °C to the laboratory for the analyses of ion chemistry and the stable isotopes of oxygen and hydrogen.

In field quality control procedures included the collection of samples in duplicates and their transportation in refrigeration. A pair of PTFE bottles with (type I) water from the Milli-Q system represented the blanks and they were carried during the entire sampling expedition to detect any possible contamination. Samples were filtered through 0.45-micron pore size nylon membranes prior to the analysis and the autosampler was rinsed with type I water to avoid cross contamination between each injection. The cation (Na^+^, Ca^2+^, Mg^2+^, K^+^ and NH_4_^+^) and anion (HCO_3_^−^, Cl^−^, SO_4_^2−^, NO_3_^−^ and PO_4_^3−^) concentrations were measured in a Waters liquid chromatograph comprising of binary pump (Model 1525), auto sampler (Model 717 plus) and conductivity detector (Model 432) as per Zamora-Martínez et al. (2016). Contents of F^−^ were estimated in an ion-selective electrode (HANAA instruments) combined with a PC 700 (Oakton) benchtop pH/conductivity meter. Six NIST certified high purity standard reference materials (i.e., IC-4–100, IC-1-A-100, IC-1–3-100, IC-1-A-100 etc.) and the certified methods under the Mexican and International norms (ISO-9001 and ISO-17025) helped in the quality control parameters. Duplicate samples were also measured after every 10 analyses, and the accuracy was evaluated with the calculation of expanded uncertainty at 95% confidence level (*U*_*exp*_). This parameter was determined for each ion considering the information generated when carrying out complete validation of the method (i.e., repeatability, reproducibility, robustness, sensitivity, selectivity, linear and working range, limits of detection and quantification) in addition to the uncertainties of the references ​​used to prepare the calibration curves. For example, the *U*_*exp*_ of Na^+^ in lake water varied between 372 and 429 mg/L and in groundwater between 0.59 and 58.07 mg/L. Similarly, the *U*_*exp*_ of Cl^−^ was 237–246 mg/L for lake water and varied between 0.14 and 71.46 mg/L for the groundwater.

Compositions of δ^18^O and δ^2^H were estimated in 4 groundwater samples from the lake proximity and all lake water samples (n = 3) in a Liquid Isotope Analyzer (Los Gatos model) using the international references of V-SMOW 2 and SLAP and the internal working standard (LGR) of Los Gatos Research (δ^18^O: 19.50 ± 0.15 ‰; δ^2^H: − 154.3 ± 0.5 ‰). The objective was to infer the influences of precipitation/evaporation and other sources of moisture recycling on the surface and groundwater chemistry and their possible interactions in the sampling season. The references and internal standard were measured in the beginning and after every 7 samples by following Coplen (2014). All results were expressed after normalizing with respect to V-SMOW, and the deuterium (d) excess was calculated as d = δ^2^H-8δ^18^O. Supplementary Table 1 presents physicochemical parameters, ion chemistry and stable isotope composition of the water samples.

## Data analysis procedures

### Drinking water quality

The drinking water suitability was evaluated by first-hand comparison of major ion concentrations with the recommended WHO guidelines (WHO, 2017). Drinking water quality index (DWQI) assessed the similarities of physicochemical parameters of water samples with WHO limits through a mathematical computation by assigning different weights (wi) and relative weights (Wi) as suggested by Jesuraja et al. (2021), Ahsan et al. (2023) and Patel et al. (2023). Critical water quality parameters, such as TDS, Cl^−^, SO_4_^2−^, NO_3_^−^ and F^−^, were allotted the maximum weight of 5, and similarly the minimum weight of 1 was assigned to HCO_3_^−^ NH_4_^+^ and PO_4_^3−^ (e.g., Tiwari et al., 2017; Vasanthavigar et al., 2010). Other parameters, such as pH (3), Ca^2+^ (3), Mg^2+^ (3), Na^+^ (4), and K^+^ (2), were assigned weights varying between 2 to 4 (Verma et al., 2020).

The relative weight (W_i_) of each parameter (pH: 0.069; TDS: 0.115; HCO_3_^−^: 0.023; Cl^−^: 0.115; SO_4_^2−^: 0.115; NO_3_^−^: 0.115; Ca^2+^: 0.069; Mg^2+^: 0.069; Na^+^: 0.092; K^+^: 0.047; F^−^: 0.115; NH_4_^+^: 0.023; PO_4_^3−^: 0.023) was calculated using the Eq. (1):

1 $${\text{Wi}} = { }\frac{{{\text{w}}_{{\text{i}}} }}{{\mathop \sum \nolimits_{{{\text{i}} = 1}}^{{\text{n}}} {\text{w}}_{{\text{i}}} }}$$

where w_i_ is the weight of each parameter and n is the number of parameters.

A quality rating scale (q_i_) for each parameter was calculated as per the Eq. (2):

2 $${\text{qi}} = { }\frac{{\left( {{\text{C}}_{{\text{i}}} } \right)}}{{\left( {{\text{S}}_{{\text{i}}} } \right)}} \times 100$$

where qi is the quality rating scale, C_i_ is concentration of each chemical parameter in each water sample in mg/L and S_i_ is the standard for each chemical parameter in mg/L as per the WHO (2017) guidelines.

Finally, the DWQI was estimated using the value of SI_*i*_ in the Eq. (3):

3 $${\text{DWQI}} = \mathop \sum \limits_{i = 1}^{n} SI_{i} ( = {\text{ w}}_{i}^{\prime } {\text{q}}_{i} )$$

where SI_i_ is the sub-index of i^th^ parameter, q_i_ is the rating based on concentration of i^th^ parameter, n is the number of parameters. It groups the samples into five different categories, such as excellent (DWQI < 50), good (DWQI: 50–100), poor (DWQI: 100–200), very poor (DWQI: 200–300) and unsuitable (DWQI > 300) for drinking as suggested by Hamma et al. (2024) and Sanad et al. (2024).

### Fluoride indigestion value

The guidelines from both Mexico (Alarcón-Herrera et al., 2020) and WHO (WHO, 2017) suggested 1.5 mg/L as the maximum allowable limit of fluoride in the drinking water. Groundwater and lake water samples were classified based on the suggested categories of fluoride ingestion as per Dissanayake (1991) and Adimalla et al. (2019b). This classification groups samples with F^−^ < 0.5 mg/L as conducive to dental caries, between 0.5 and 1.5 mg/L as favorable for good dental and skeletal health, 1.5–4 mg/L as promotor of dental fluorosis in children, 4–10 mg/L as promotor of both dental and skeletal fluorosis and > 10 mg/L as possible health risk from crippling skeletal fluorosis.

### Nitrate pollution index

In the context of WHO guidelines for drinking water quality, the maximum acceptable value of nitrate is 50 mg/L (WHO, 2017). The levels of pollution were estimated by calculating the Nitrate Pollution Index (NPI) with the following formula (e.g., Panneerselvam et al., 2020; Li et al., 2023; Karunanidhi et al., 2024).

$${\text{NPI}} = { }\frac{{{\text{Cs}} - {\text{TVN}}}}{{{\text{TVN}}}}$$

where Cs represents the nitrate content in water and TVN is the threshold value of nitrate.

Nitrate content up to 10 mg/L is considered natural and above it indicates contamination (Xiao et al., 2022). Therefore, the TVN value to calculate NPI is represented here by 10 mg/L (Das et al., 2023). NPI categorizes the samples as unpolluted or clean (NPI < 0), slightly polluted (NPI: 0–1), moderately polluted (NPI: 1–2), significantly polluted (NPI: 2–3) and very significantly polluted (NPI > 3).

### Health risk assessment

The health risk is estimated from intake of F^−^ and NO_3_^−^ through the drinking water pathway for the more vulnerable population groups in the region by assessing the impacts on 6-month infant, 11-year-old child, 78-year older adult and 45-year elderly pregnant woman by following the recommendation of United States Environmental Protection Agency (USEPA, 2004). The non-carcinogenic risk for each age group was estimated by calculating the average daily exposure dose (DE) through ingestion of groundwater and lake water [mg/kg/day] by using the formula of USEPA (2004) (e.g., Adimalla et al., 2019a; Karunanidhi et al., 2020; Subba Rao et al., 2020; Sabino et al., 2022):

$${\text{DE}} = \frac{{{\text{C}}_{{\text{P}}} \times {\text{IR}} \times {\text{ED}} \times {\text{EF}}}}{{{\text{AB}} \times {\text{AE}}}}{ }$$

where C_P_ is pollutant concentration (mg/L), IR is ingestion rate per unit time (infant: 1.104 L/day; children: 1.258 L/day; older adults: 3.229 L/day and pregnant women: 2.935 L/day; USEPA analysis 2019 data at 95th percentile (ATSDR, 2023)), ED is exposure (year) duration (infant: 0.5; children: 11; older adults: 78: pregnant women: 45 years; ATSDR, 2023), and EF is exposure frequency (365 days/year; Subba Rao et al., 2020). We considered the average body weight (AB) as recommended by the Agency for Toxic Substances and Disease Registry with 7.4 kg for infants, 31.8 kg for 11-year child, 80 kg for older adult and 73 kg for pregnant woman. AE is the average age (day) exposure time (infant: 2701; child: 11,606; older adult: 29,198 and pregnant woman: 26,644 days; ATSDR, 2023).

The hazard quotient (HQ) was applied to evaluate risk from the consumptions of fluoride and nitrate in water samples using the equation:

$${\text{HQ}} = \frac{{{\text{DE}}}}{{{\text{RfD}}}}{ }$$

where HQ is the hazard quotient for fluoride and nitrate, and it measures the non-carcinogenic chronic hazard, and RfD represents the reference dose for chronic oral exposure (mg/kg/day) to both the pollutants (F^−^: 0.06 mg/ kg/day; NO_3_^−^: 1.60 mg/ kg/day; Alarcón-Herrera et al., 2020). Finally, the Total Hazard Quotient Index (THQI) of non-carcinogenic risk was evaluated by adding the values of HQ_flouride_ and HQ_nitrate_:

$${\text{THQI }} = {\text{ HQ}}_{{{\text{fluoride}}}} + {\text{ HQ}}_{{{\text{nitrate}}}}$$

The samples with HQ and THQI above 1 may cause non-carcinogenic risk and they are considered inappropriate for ingestion. Samples with HQ and THQI below 1 are considered safe for consumption (e.g., He et al., 2019; Sabino et al., 2022).

## Results and discussion

### Geochemical process and facies

#### Groundwater

**Fig. 2:**
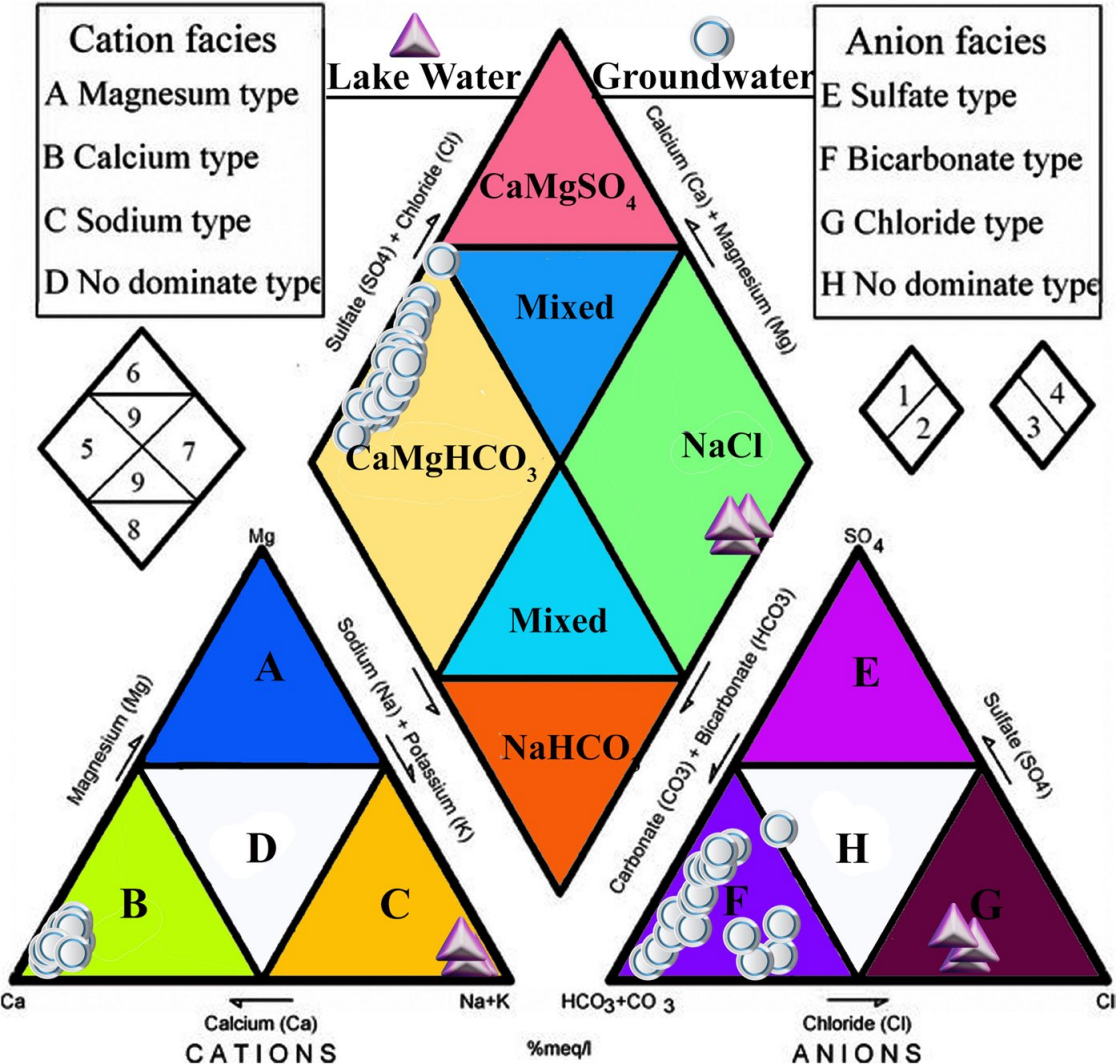
A trilinear Piper diagram showing the hydrochemical facies of groundwater and lake water samples from the Oriental Basin in eastern-central Mexico

**Fig. 3:**
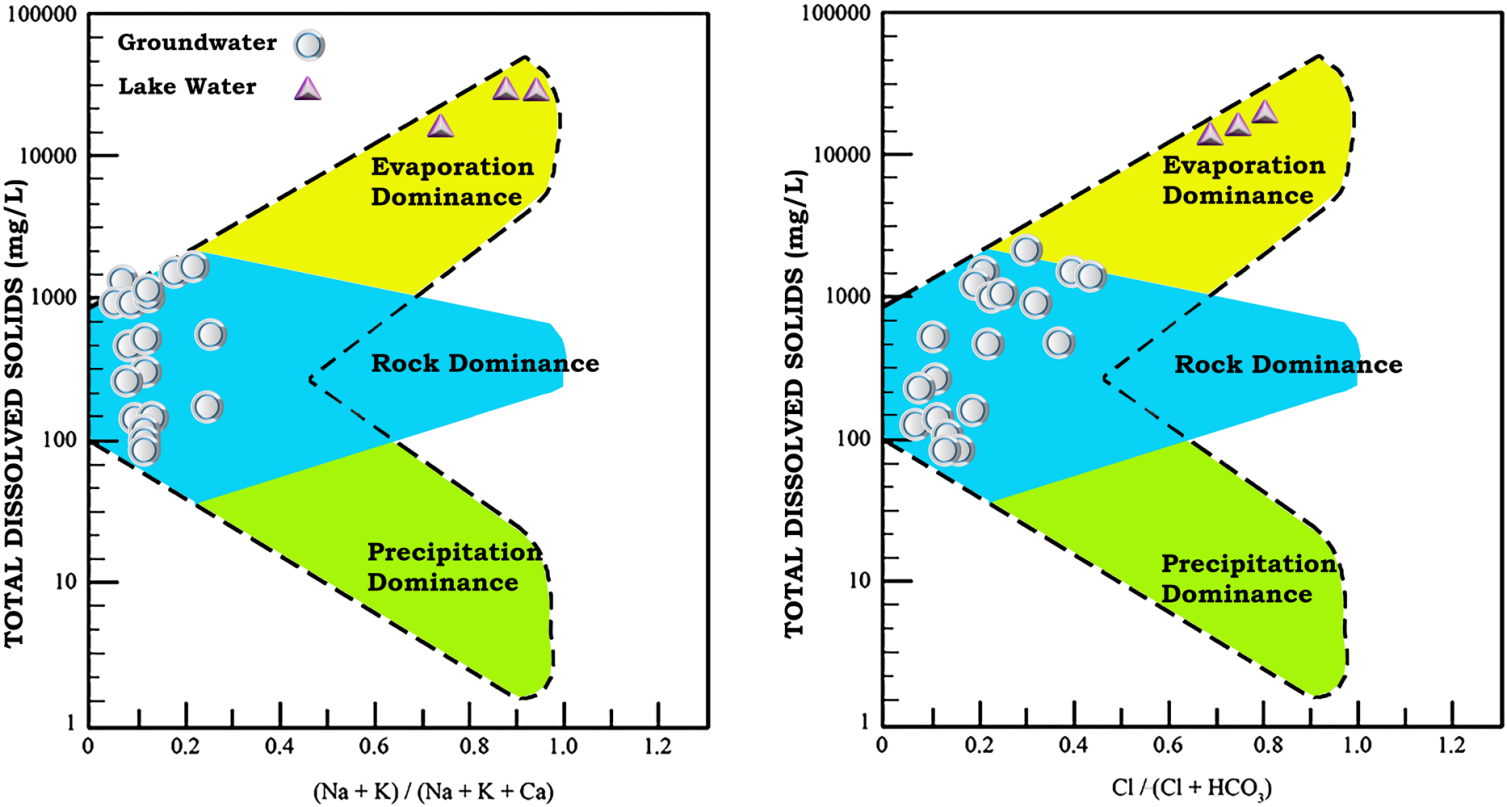
Gibbs diagrams showing the influences of precipitation, evaporation, and rock-water interaction on hydrochemistry of groundwater and lake water from the Oriental Basin in eastern-central Mexico

Groundwater (n = 20) with variable pH (6.5–9.1) and TDS (145–1270 mg/L) showed more heterogeneity in a Piper trilinear plot (Table 1, Fig. 2). The samples were classified as Ca-Mg-HCO_3_ facies with the anion triangle showing dominant HCO_3_-type and the cation triangle indicating Ca type (Fig. 2). This chemical facies mirrors interactions with limestone in deeper part of the aquifer and Ca-bearing (i.e., feldspars) volcanic lithologies, such as basalt and andesite, in the shallow part (e.g., Servicio Geologico Mexicano, 2011a, 2011b, 2012; CONAGUA, 2023). The organic matter oxidation in topsoil might have also contributed additional HCO_3_^−^. Interactions with the limestone, volcanic deposits and organic matter-bearing topsoil influenced the chemical facies (e.g., Eugster & Hardie, 1978). Generally lower Na + K/(Na + K + Ca) values in Gibbs diagrams, however, reflected the dominant interaction with limestone (Fig. 3; Marandi & Shand, 2018). Higher Cl/Cl + HCO_3_ in some samples could be indicative of the irrigation return-flow causing Cl^−^ enrichment (e.g., Park et al., 2018). The samples with Cl^−^ up to 262.3 mg/L were collected from wells near the agricultural fields. Additional evidence of the return flow could be the higher nitrate in wells with lower static levels and lower nitrate contents in wells with higher static levels. For example, the well COR-2 with NO_3_^−^ > 50 mg/L has a static level of ca.10 m and the well COR-16 with nitrate below the detection limit has a static level of ca.30 m (Fig. 1A).

**Table 1:** Summarized physico-chemical parameters of groundwater and water from the Totolcingo Lake in Oriental Basin (eastern-central Mexico) with respect to WHO (2017) recommended values

	Groundwater (n = 20)			Lake water (n = 3)			WHO (2017)
	Min–Max	Avg	> permissible limit, number (%)	Min–Max	Avg	> permissible limit, number (%)	
pH	6.5–9.1	7.5	1 (5%)	9.6–9.7	9.7	3 (100%)	6.5–8.5
EC (µS/cm)	290–2560	1265	9 (45%)	40,200–44,200	42,033	3 (100%)	1500
TDS (mg/L)	145–1270	632	5 (25%)	20,100–22100	20,900	3 (100%)	1000
Ca^2+^ (mg/L)	21.1–242.5	112.3	9 (45%)	3.5–4.2	3.9	0 (0%)	75
Mg^2+^ (mg/L)	8.8–154.6	65.1	11 (55%)	4.0–7.3	5.2	0 (0%)	50
Na^+^ (mg/L)	17–268.6	73.2	1 (5%)	10,679–12300	11,422	3 (100%)	200
K^+^ (mg/L)	2.9–20.3	8.7	4 (20%)	1377–1549	1439	3 (100%)	12
HCO_3_^−^ (mg/L)	128–1345.9	623.8	13 (65%)	12,137–14,823	13,516	3 (100%)	300
Cl^−^ (mg/L)	4.3–262.3	82.2	1 (5%)	6904–7662	7316	3 (100%)	250
SO_4_^2−^ (mg/L)	3.4–374.9	102.3	3 (15%)	3808–4312	3992	3 (100%)	250
NO_3_^−^ (mg/L)	< LD (0.75)–75.3	14.1	2 (10%)	< LD (0.75)	–	–	50
F^−^ (mg/L)	2.5–9.9	6.5	20 (100%)	12.7–13.2	13.0	3 (100%)	1.5
NH_4_^+^ (mg/L)	< LD (0.5)–7.9	2.1	7 (35%)	< LD (0.5)	–	–	1.5
PO_4_^3−^ (mg/l)	< LD (0.75)–52.5	7.2	13 (65%)	102–126	114	3 (100%)	0.5

Sample numbers and sample % above the permissible limits for each parameter are presented (LD: limit of detection)

**Fig. 4:**
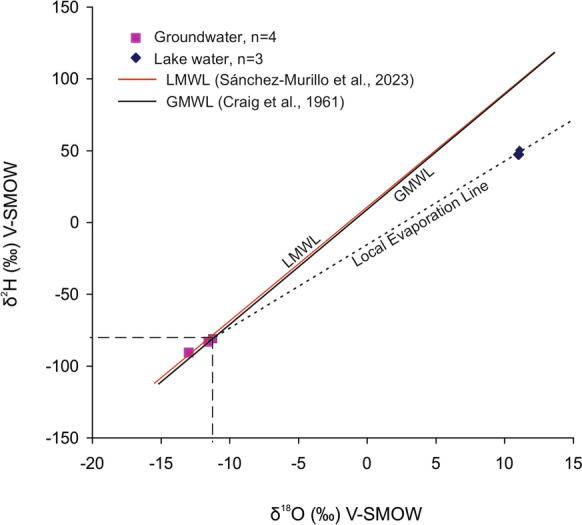
Distributions of δ^18^O_H2O_ vs. δ^2^H_H2O_ in groundwater (n = 4) and water from the Totolcingo Lake water (n = 3) in the Oriental Basin of eastern-central Mexico with respect to the local meteoric water line (LMWL; Sánchez-Murillo et al., 2023) and global meteoric water line (GMWL; Craig, 1961). The local meteoric water line represented precipitation at Veracruz (Mexico) between 2018 and 2021

The ranges of δ^18^O (− 12.97‰ to − 7.00‰) and δ^2^H (− 90.60‰ to − 56.10‰) located the groundwater samples along tendencies of the global meteoric water line (GMWL, Craig, 1961) and a local meteoric water line (LMWL, Sánchez-Murillo et al., 2023), representing the precipitation (δ^2^H = 7.9δ^18^O + 11.2, n = 38, 2018–2021) at Veracruz, i.e., ca. 155 km east of the Oriental Basin at the Gulf of Mexico coast (Fig. 4). Except for one sample (− 0.10‰), the d-excess values (8.90–13.20‰) were comparable to the average global precipitation (+ 10‰; Clark & Fritz, 1997) as well as local precipitation (+ 11.2‰; Sánchez-Murillo et al., 2023). Similar to the observations in Gibbs diagram, the stable isotope characteristics (δ^18^O, δ^2^H and d-excess) did not indicate the influence of evaporation on the groundwater chemistry. Variation of ca. 4‰ in d-excess reflected the moisture recycling on precipitation through the terrestrial evapotranspiration, sub-cloud raindrop re-evaporation, net rainout, recharge region altitude and sea-surface temperature (Clark & Fritz, 1997; Cui et al., 2017; Natali et al., 2022; Pfahl & Sodemann, 2014; Wassenaar et al., 2009; Xia & Winnick, 2021).

#### Lake water

The lake water (n = 3), characterized by alkaline pH (9.6–9.7, Table 1) and very high TDS (20,100–22,100 mg/L), were homogeneous in the trilinear Piper (1944) plot and represented the Na-Cl facies (Fig. 2). Distributions of cation classified them as Na-type and the anions grouped them as Cl-type. Both the Gibbs (1970) plots suggested influence of evaporation on the hydrochemistry (Fig. 3). Enrichment of heavy isotopes (δ^18^O: 10.98–11.08‰; δ^2^H:47.4–50.4‰) through extreme evaporation in Totolcingo Lake was also supported by the physical parameter and ion compositions. The lake water showed several fold higher salinities and concentrations of Na^+^, Cl^−^ and SO_4_^2−^ compared to the groundwater (see Table 1). For example, the average Na^+^ and Cl^−^ concentrations in lake water were 156-fold and 89-fold higher than the groundwater, respectively. The average TDS of lake water was 33-fold higher compared to the groundwater. The lake water samples on a local evaporation line and their depleted d-excess values (− 40.80 to − 38.20‰) compared to the precipitation (GMWL and LMWL) and groundwater also reflected the dominance of evaporation on surface water (Fig. 4 and Table 1). Similar to this study, the water from Cona Lake in Tibetan Plateau with depleted mean d-excess (− 7.5‰) reflected the influence of evaporation (Cui et al., 2017). The depleted d-excess (− 40.05‰ to − 17.99‰) in water from the Lake Coatetelco (Mexico) also reflected variable degrees of evaporation in different seasons (Roy et al., 2024). Deviation of lake water with enriched δ^18^O and δ^2^H from the groundwater suggested lack of surface water and groundwater interaction during the sampling season (i.e., prior to the rainy summer-autumn). It is also supported by their significantly different physicochemical characteristics (Table 1). The chemical facies of lake water (Na-Cl) and groundwater (Ca-Mg-HCO_3,_ Fig. 2) are also different.

### Drinking water suitability

#### Groundwater

Generally, the pH (6.5–9.1) remained within the permissible limit for drinking as per the WHO guidelines, except for one well (Table 1). EC (290–2560 μS/cm) above the WHO recommended value (> 1500 μS/cm) in 45% samples and TDS (145–1270 mg/L) above the WHO permissible limit (> 1000 m/L) in 25% of samples showed more dissolved ions from the rock-water interaction. Compared to the observations of Can-Chulim et al. (2011), the groundwater of this study was relatively more acidic with an average pH of 7.5 and more conductive with an average EC of 1265 μS/cm. In the groundwater samples collected in March 2008 from 25 wells in the Oriental Basin, Can-Chulim et al. (2011) reported pH between 7.8 and 9.1 and EC between 190 and 1833 μS/cm.

About 65% samples also had HCO_3_^−^ (128–1345.9 mg/L), 55% samples had Mg^2+^ (8.8–154.6 mg/L), and 45% samples had Ca^2+^ (21.1–242.5 mg/L) above the WHO recommended values (Aragaw & Gnanachandrasamy, 2021; Badeenezhad et al., 2020). More bicarbonate, calcium and magnesium in these samples showed the effect of limestone dissolution as well as chemical weathering of volcanic lithologies (e.g., Chung et al., 2020; Eugster & Hardie, 1978). PO_4_^3−^ with concentrations up to 52.5 mg/L and 65% samples above the permissible value (> 0.5 mg/L) reflected attribution of different anthropogenic activities, such as the application of fertilizers (including the extensive usage of poultry manure) and pesticides in agriculture and the discharge of domestic sewage and industrial wastewater (e.g., Huang et al., 2020; Zhou et al., 2022; Table 1). Dissolution of phosphate-bearing minerals (fluorapatite, hydroxyapatite and carbonate-fluorapatite) as well as the influence of alluvial-lacustrine sediments containing peat and organic matter might also have contributed some of the phosphate (e.g., Fayiga & Nwoke, 2016; Liu et al., 2020; Zhou et al., 2022). Concentration of NH_4_^+^ up to 7.9 mg/L (> 1.5 mg/L in 35% samples) was not directly relevant but could lead to free chlorine reduction and chloramines formation (WHO, 2017).

**Fig. 5:**
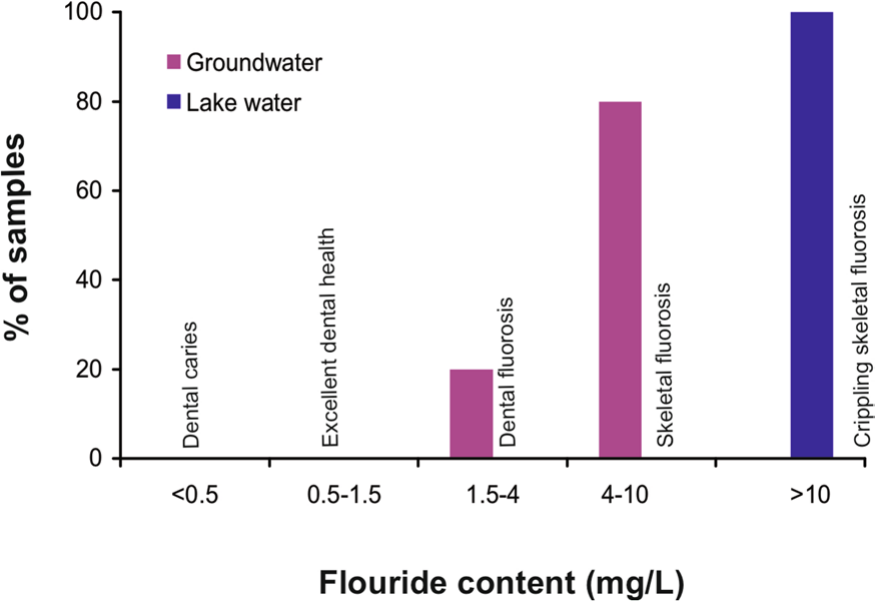
Classification (%) of groundwater and lake water samples from the Oriental Basin in eastern-central Mexico based on the health effects of fluoride ingestion (e.g., Adimalla et al., 2019b; Dissanayake, 1991)

Between 5 and 20% samples had Na^+^ (17–268.6 mg/L), K^+^ (2.9–20.3 mg/L), Cl^−^ (4.3–262.3 mg/L) and SO_4_^2−^(3.4–374.9 mg/L) above the permissible limits. All the samples (100%, n = 20), however, had fluoride (2.5–9.9 mg/L) above the recommended 1.5 mg/L. Based on the classification of Dissanayake (1991) and subsequently used in case studies by Adimalla et al. (2019b), about 20% groundwater samples (n = 4) from the Oriental Basin were categorized as possible promotor of dental fluorosis (F: 1.5–4 mg/L) and 80% (n = 16) samples as promotor of both dental and skeletal fluorosis (F: 4–10 mg/L) (Table 2, Fig. 5). The groundwater of Zacatepec aquifer in central-southern Mexico (region IV, Balsas) with up to 8.7 mg/L of F^−^ and in the Chihuahua state with up to 6 mg/L of fluoride were also promotors of both dental and skeletal fluorosis (Reyes-Gomez et al., 2017; Roy et al., 2023).

**Table 2:** Classification of fluoride contents in groundwater and water from the Totolcingo Lake (Oriental Basin, eastern-central Mexico) based on human health effects (Adimalla et al., 2019a, 2019b; Dissanayake, 1991)

Fluoride concentration	Effect on human health	Groundwater, n = 20 number (%)	Lake water, n = 3 number (%)
< 0.5 mg/L	Conducive to dental caries	–	–
0.5–1.5 mg/L	Promotes excellent dental health	–	–
1.5–4.0 mg/L	Dental fluorosis in children	4 (20%)	–
4.0–10.0 mg/L	Dental and skeletal fluorosis	16 (80%)	–
> 10.0 mg/L	Crippling skeletal fluorosis	–	3 (100%)

**Fig. 6:**
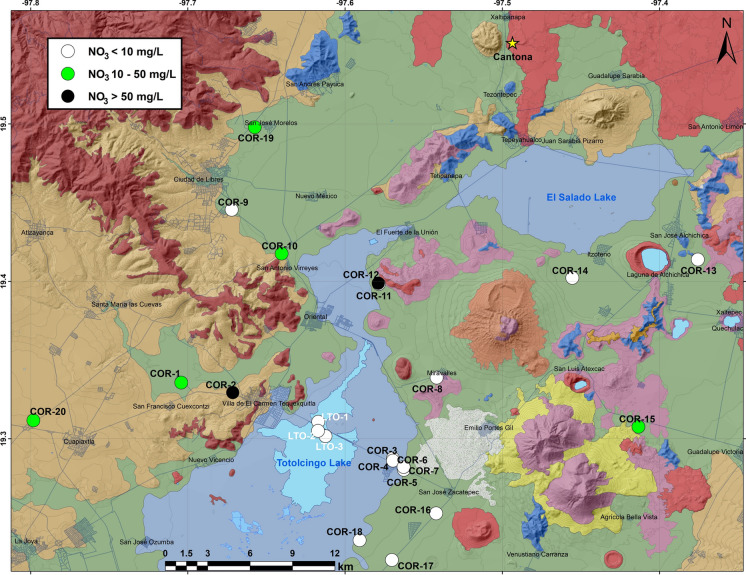
Distribution of groundwater wells and water samples from the Totolcingo Lake (Oriental Basin, eastern-central Mexico) with NO_3_ < 10 mg/L, NO_3_ between10 and 50 mg/L and NO_3_ > 50 mg/L

NO_3_^−^ remained above the permissible limit of WHO (> 50 mg/L) in only 10% samples (Fig. 6). It is a common anthropogenic pollutant sourced from the application of fertilizers, and the irrigation by sewage effluents and unsewered sanitation (Badeenezhad et al., 2021; Jia et al., 2023; Karunanidhi et al., 2024; Naik et al., 2022). Abascal et al. (2022) observed that agriculture, industry, and sewage are the main contributors of NO_3_^−^ into the water resources of Mexico. The samples with nitrate < 10 mg/L (n = 13) showed natural conditions, with minimal human interferences, and the samples with nitrate between 10 and 50 mg/L (n = 5) at western part of the basin, around some of the villages, indicated anthropogenic impact possibly from poorly treated domestic discharge and sewage leakage into the aquifer (Fig. 6, e.g., Marghade et al., 2015; Subba Rao et al., 2020). Torres-Martínez et al. (2021) reported NO_3_^−^ up to 109 mg/L in groundwater from the Comarca Lagunera (northern Mexico) and revealed that the application of manure contributed about 48%, urban sewage provided ca. 43% and synthetic fertilizers contributed 5% of the total nitrate contents.

Nitrate Pollution Index (NPI) categorized 65% (n = 13) of the groundwater samples as unpolluted or clean, 5% (n = 1) as slightly polluted, 20% (n = 4) as moderately polluted with NPI of 1–2 and 10% (n = 2) as very significantly polluted with NPI > 3 (Table 3, e.g., Panneerselvam et al., 2021; Karunanidhi et al., 2024). Different degrees of nitrate pollution in 35% samples could be from the application of inorganic nitrogenous fertilizers and manures in the agricultural activity, influence from wastewater disposal and oxidation of human and other animal excreta in the septic tanks. Negative correlation between NO_3_^−^ and Cl^−^ (r = − 0.5), however, suggested more influence of synthetic fertilizers (e.g., ammonium nitrate) compared to the sewage leakages (e.g., Mahlknecht et al., 2008). The molar ratios of NO_3_/Cl vs. Cl^−^ concentration (μmol) also helped to identify the nitrate sources. Low NO_3_/Cl ratio with high Cl^−^ concentration characterizes the influences of manure and sewage, whereas the high NO_3_/Cl ratio with low Cl^−^ concentration shows more contribution from the chemical fertilizer (Jung et al., 2023; Sánchez-Gutiérrez et al., 2023). The groundwater samples with different NPI values showed highly variable Cl^−^ (121–2700 μmol) and NO_3_/Cl ratios (0.2–9.4). Both suggested the influences of fertilizer as well as the manure and sewage, as this rural part of Mexico lacks adequate sanitization management. Both the wells (COR-2 and COR-12, Fig. 1A) showing very significant nitrate pollution (NPI > 3) are located close to the agricultural activities (Fig. 1B, C). The industrial activity in Oriental Basin is represented by pig farming, poultry farming, food processing, and breweries and some of them might have also released synthetic chemicals with high nitrogen and phosphorus into the groundwater (Source: La Jornada de Oriente, March 15, 2021). Torres-Martínez et al. (2020) have associated nitrate pollution in the urban areas of Monterrey (northern Mexico) with the sewage leakage and Rojas-Fabro et al. (2015) associated the above permissible nitrate in groundwater of the Merida City (south-east Mexico) to the domestic wastewater and agricultural activity.

**Table 3:** Classification of Nitrate Pollution Index (NPI; Panneerselvam et al., 2021) values of groundwater and water from the Totolcingo Lake (Oriental Basin, eastern-central Mexico)

NPI value	Class	Groundwater, n = 20 number (%)	Lake water, n = 3 number (%)
< 0	Unpolluted or clean	13 (65%)	3 (100%)
0–1	Slightly polluted	1 (5%)	–
1–2	Moderately polluted	4 (20%)	–
2–3	Significantly polluted	–	–
> 3	Very significantly polluted	2 (10%)	–

NPI of lake water was < 0 as nitrate remained below the detection limit (< 0.75 mg/L)

The heterogenous DWQI values (28–282, avg: 105) grouped about 30% samples (n = 6) in poor category and 10% samples (n = 2) in very poor category for drinking (Table 4). Only one sample (5%) was in excellent category with DWQI < 50 and 55% (n = 11) samples were in the good category (Table 4). The well with excellent drinking water quality (COR-9, Fig. 1), however, contained F^−^ (9.9 mg/L) above the WHO guideline. The wells with good quality drinking water (COR-1, 5, 6, 7, 10, 13, 15, 16, 17, 19 and 20) contained NO_3_^−^ below the WHO permissible value (< 50 mg/L) but fluoride higher than the permissible 1.5 mg/L. The poor and very poor drinking water wells (COR-2 and 12) were characterized by fluoride (9.8 mg/L, COR-18) as well as nitrate (> 50 mg/L) above the permissible values.

**Table 4:** Classification of drinking water quality index (DWQI) values estimated for groundwater and water from the Totolcingo Lake (Oriental Basin, eastern-central Mexico)

DWQI value	Categories	Groundwater, n = 20 number (%)	Lake water, n = 3 number (%)
< 50	Excellent water	1 (5%)	–
50–100	Good water	11 (55%)	–
100–200	Poor water	6 (30%)	–
200–300	Very Poor water	2 (10%)	–
> 300	Unsuitable for drinking	–	3 (100%)

#### Lake water

About 60.6% of the total water consumption in Mexico is sourced from surface water bodies. Even through the physicochemical characteristics of lake water in the sampling season were unsuitable, we evaluated it with the parameters of groundwater in order to contribute new quality monitoring data from a surface water body. This evaluation is useful in a regional context as the water quality of Lake Cajititlán at western Mexico is also affected by excess fertilizer runoff and poorly treated wastewater (Gradilla-Hernández et al., 2020) and the Lake Coatetelco in central-southern Mexico contained more than the permissible fluoride (Roy et al., 2023). The physical parameters (pH: 9.6–9.7; EC: 40,200–44,200 μS/cm; TDS: 20,100–22,100 mg/L) and ion concentrations (Na^+^: 10,679–12,300 mg/L; K^+^: 1377–1549 mg/L; HCO_3_^−^: 12,137–14,823 mg/L; Cl^−^: 6904–7662 mg/L; SO_4_^2−^: 3808–4312 mg/L) of water from the Totolcingo Lake remained above the WHO recommendations (Table 1; Aragaw & Gnanachandrasamy, 2021; Badeenezhad et al., 2020). DWQI values (2540–2677; avg: 2591) categorized all the samples as unsuitable (Table 4). Fluoride (12.7–13.3 mg/L) contents were comparable to the water resources showing possible risk from the crippling skeletal fluorosis (Table 2 and Fig. 5, Dissanayake, 1991; Adimalla et al., 2019b). The correlations of F^−^ with TDS (r = 0.8) and Na^+^ (r = 0.6) suggested that the influence of evaporation in enriching fluoride (Adam et al., 2001; Rafique et al., 2015; Shaji et al., 2024). Enrichment of ions and physical parameters also indicated the effect of evaporation (e.g., Chung et al., 2020; Eugster & Hardie, 1978). More evaporation of the lake water is reflected by enriched values of heavier isotopes of oxygen and hydrogen and their distributions along the local evaporation line (Fig. 4). Both fluoride and calcium contents were negatively correlated (r = − 0.3) in this study. It is reflected by less Ca^2+^ (3.5–4.2 mg/L) in lake water compared to the groundwater (Ca^2+^: 21.1–242.5 mg/L, Table 1). Both Handa (1975) and Adimalla et al. (2019b) have also noted fluoride enrichment in the calcium-deficient water. Above permissible PO_4_^3−^ (102–126 mg/L) reflected the pollutant enrichment through evaporation of the surface water (Table 1). NPI < 0, however, suggested loss of N possibly through the processes of ammonia volatilization and formation of gaseous NH_3_ in an alkaline environment (Leng et al., 2006). Negligible nitrate and ammonium contents (below the detection limits) in the Lake Totolcingo with pH of 9.6–9.7 supported both the processes.

### Health risk assessment

**Fig. 7:**
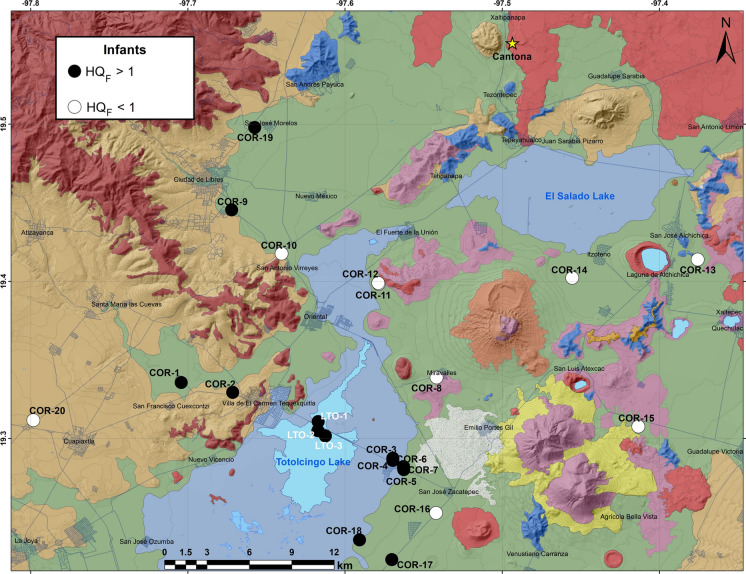
Distribution of groundwater wells and water samples from the Totolcingo Lake (Oriental Basin, eastern-central Mexico) with HQ_fluoride_ > 1 for infants

The ingestion of water with fluoride > 1.5 mg/L triggers dental and skeletal fluorosis, and concentrations of nitrate above 50 mg/L causes methemoglobinemia in infants (Adimalla et al., 2019a; Karunanidhi et al., 2020; Kimambo et al., 2019; Alarcón-Herrera et al., 2020). We computed non-carcinogenic risks through HQ_fluoride_ and HQ_nitrate_ for different age groups residing in the Oriental Basin (Mexico) from fluoride and nitrate consumptions through the drinking water by considering the average weights of an infant as 7.4 kg, 11-year-old child as 31.8 kg, an older adult (78 year) as 80 kg and an elderly pregnant woman (45 year) as 73 kg in Table 5. HQ_fluoride_ > 1 showed potential fluorosis risk for the older adults and pregnant women from all the groundwater samples (100%, n = 20). The health risk for children could be from 80% (n = 16) of them and about 55% of groundwater wells (n = 11), mostly at western and southern parts of the basin pose fluorosis risk to the infants (Fig. 7). This is in congruence with the last national dental and caries and fluorosis survey (2011–2014) involving about 74,000 students of 6, 9, 12 and 15 years from 32 different federal entities of Mexico (ENCD, 2018). The dental fluorosis in Puebla state was relatively higher than other federal entities with the prevalence level of 2.6% in 6 years old. The prevalence was much higher for the 12 and 15-year-old children with 51.6% and 60.4%, respectively. Both the later groups had permanent dental caries in an average 1.4 and 2.2 teeth, respectively (ENCD, 2018). HQ_fluoride_ influenced the Total Health Index (THQI) values and it suggested potential non-carcinogenic concerns in the order of older adults (1.63–6.50; avg: 4.30) > elderly pregnant women (1.03–4.09, avg: 2.71) > children (0.57–2.26; avg: 1.49) > infants (0.42–1.66; avg: 1.10) from F^−^ enrichment in groundwater (Table 5). Higher fluoride in water samples of this study could be from weathering of fluoride-bearing minerals through the rock-water interactions. Shaji et al. (2024) suggested that the weathering of fluorite, cryolite, fluocerite, yttrofluorite, villianmite, sellaite, fluorapatite, and the volcanic deposits contribute F^−^ into the groundwater. Positive correlations of F^−^ with pH (r = 0.6) and HCO_3_^−^ (r = 0.7) showed that the alkaline environment facilitated mobility of fluoride through the source rock leaching (Alvarez & Carol, 2019; Crundwell, 2017; Shaji et al., 2024). Thus, its sustainable management would require regular water quality monitoring and generation of maps indicating F^−^ concentrations, application of disinfection techniques such as reverse osmosis and a detailed study of F-bearing minerals in different volcanic lithologies as well as limestones present in the watershed and their fluoride contents.

**Table 5:** Computed HQ and THQI values for fluoride and nitrate in groundwater and water from the Totolcingo Lake (Oriental Basin, eastern-central Mexico) for infants, children, older adults and vulnerable pregnant women with average weights of 7.4 kg, 31.8 kg, 80 kg and 73 kg, respectively

Sample	Hazard Quotient (HQ_fluoride_)				Hazard Quotient (HQ_nitrate_)				THQI			
	Infant	Children	Older adult	Pregnant woman	Infant	Children	Older adult	Pregnant woman	Infant	Children	Older adult	Pregnant woman
*Ground water*												
COR-1	**1.31**	**1.78**	**5.12**	**3.22**	0.13	0.17	0.50	0.31	**1.44**	**1.95**	**3.72**	**3.53**
COR-2	**1.05**	**1.43**	**4.11**	**2.59**	0.45	0.61	**1.74**	**1.10**	**1.50**	**2.04**	**4.33**	**3.69**
COR-3	**1.33**	**1.81**	**5.20**	**3.27**	–	–	–	–	**1.33**	**1.81**	**3.27**	**3.27**
COR-4	**1.42**	**1.93**	**5.56**	**3.50**	–	–	–	–	**1.42**	**1.93**	**3.50**	**3.50**
COR-5	**1.42**	**1.92**	**5.53**	**3.48**	–	–	–	–	**1.42**	**1.92**	**3.48**	**3.48**
COR-6	**1.30**	**1.77**	**5.09**	**3.21**	–	–	–	–	**1.30**	**1.77**	**3.21**	**3.21**
COR-7	**1.49**	**2.03**	**5.83**	**3.67**	–	–	–	–	**1.49**	**2.03**	**3.67**	**3.67**
COR-8	0.95	**1.30**	**3.73**	**2.35**	–	–	–	–	0.95	**1.30**	**2.35**	**2.35**
COR-9	**1.66**	**2.26**	**6.50**	**4.09**	0.05	0.07	0.21	0.13	**1.72**	**2.33**	**4.31**	**4.23**
COR-10	0.95	**1.29**	**3.71**	**2.33**	0.12	0.16	0.46	0.29	**1.07**	**1.45**	**2.80**	**2.63**
COR-11	0.97	**1.32**	**3.79**	**2.38**	–	–	–	–	0.97	**1.32**	**2.38**	**2.38**
COR-12	0.85	**1.16**	**3.32**	**2.09**	0.47	0.64	**1.85**	**1.17**	**1.33**	**1.80**	**3.95**	**3.26**
COR-13	0.46	0.63	**1.80**	**1.13**	0.05	0.06	0.18	0.11	0.51	0.69	**1.31**	**1.25**
COR-14	0.42	0.57	**1.63**	**1.03**	–	–	–	–	0.42	0.57	**1.03**	**1.03**
COR-15	0.50	0.68	**1.97**	**1.24**	0.16	0.22	0.64	0.40	0.67	0.91	**1.88**	**1.64**
COR-16	0.48	0.66	**1.89**	**1.19**	–	–	–	–	0.48	0.66	**1.19**	**1.19**
COR-17	**1.51**	**2.05**	**5.89**	**3.71**	–	–	–	–	**1.51**	**2.05**	**3.71**	**3.71**
COR-18	**1.65**	**2.24**	**6.43**	**4.05**	–	–	–	–	**1.65**	**2.24**	**4.05**	**4.05**
COR-19	**1.30**	**1.76**	**5.06**	**3.19**	0.17	0.23	0.67	0.42	**1.47**	**1.99**	**3.86**	**3.61**
COR-20	0.96	**1.31**	**3.77**	**2.37**	0.17	0.23	0.67	0.42	**1.14**	**1.54**	**3.04**	**2.79**
Minimum	0.42	0.57	1.63	1.03	0.05	0.06	0.18	0.11	0.42	0.57	1.03	1.03
Maximum	1.66	2.26	6.50	4.09	0.47	0.64	1.85	1.17	1.72	2.33	4.33	4.23
Average	1.10	1.49	4.30	2.71	0.09	0.12	0.35	0.22	1.19	1.61	3.05	2.92
*Lake water*												
LTO-1	**2.18**	**2.96**	**8.51**	**5.36**	–	–	–	–	**2.18**	**2.96**	**8.51**	**5.36**
LTO-2	**2.14**	**2.90**	**8.34**	**5.25**	–	–	–	–	**2.14**	**2.90**	**8.34**	**5.25**
LTO-3	**2.23**	**3.02**	**8.69**	**5.47**	–	–	–	–	**2.23**	**3.02**	**8.69**	**5.47**
Minimum	2.14	2.90	8.34	5.25	–	–	–	–	2.14	2.90	8.34	5.25
Maximum	2.23	3.02	8.69	5.47	–	–	–	–	2.23	3.02	8.69	5.47
Average	2.18	2.96	8.51	5.36	–	–	–	–	2.18	2.96	8.51	5.36

Bold indicates HQ > 1

**Fig. 8:**
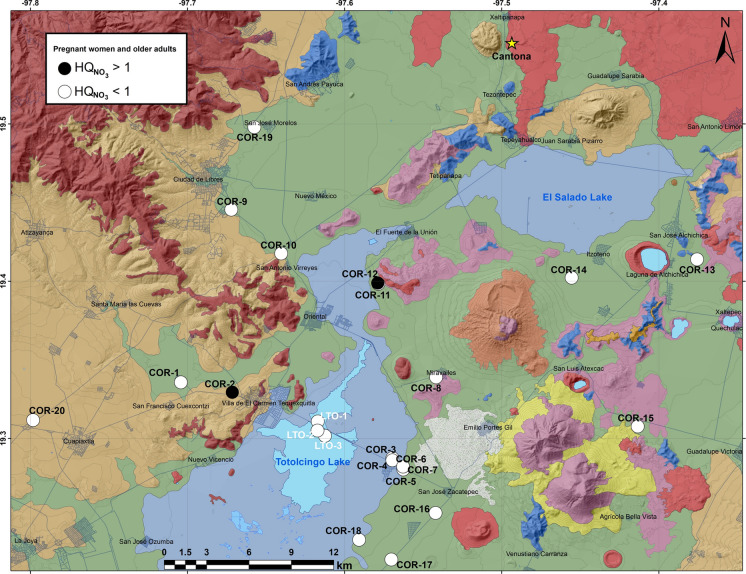
Distribution of groundwater wells and water samples from the Totolcingo Lake (Oriental Basin, eastern-central Mexico) with HQ_nitrate_ > 1 for pregnant women and older adults

HQ_nitrate_ could not be computed in some groundwater samples as nitrate remained below the detection limit (< 0.75 mg/L). It suggested more non-carcinogenic concerns for older adults (0.18–1.85; avg: 0.35) and elderly pregnant women (0.11–1.17, avg: 0.22) compared to the infants (0.05–0.47; avg: 0.09) and children (0.06–0.64; avg: 0.12; Table 5). Health threat for the adult population was caused from only 10% samples (n = 2) and it was due to their higher ingestion rates (Fig. 8). None of the groundwater sample exposed the infants to possible methaemoglobinaemia and most of the samples also did not show any immediate health risk for the other groups. However, these results showed the necessity to identify the nitrate pollution sources, and regulation of fertilizer application and sewage leakages in this region. The presence of ammonia in some groundwater samples (n = 4, see supplementary Table 1) possibly can enhance the nitrate concentration by nitrification (WHO, 2017). Similarly, the boiling of drinking water to ensure microbiological safety could enhance the nitrate content (10–50 mg/L) in water from 5 wells (COR-1, 10, 15, 19 and 20) at western part of the basin beyond the WHO limit, and might cause potential health risks for the infants (e.g., Alvarado et al., 2022; WHO, 2017). Water from the wells with NO_3_^−^ above 10 m/L might require disinfection through the ion exchange, reverse osmosis, biological denitrification and electrodialysis to reduce the risk of gastrointestinal infection and methaemoglobinaemia (WHO, 2017). Authorities should monitor the quality of water used by the bottle-fed infants, especially those with symptoms of gastrointestinal infection such as diarrhea. The mothers and expectant mothers should be receiving information about water safety, guidance on disinfection and access to the safe water sources.

## Conclusions

In the Oriental Basin of eastern-central Mexico, the physical and chemical characteristics along with stable isotopes of oxygen and hydrogen in water from wells and Totolcingo Lake helped to evaluate their suitability for drinking. The health risks assessment through the fluoride and nitrate consumptions in different vulnerable age groups provided a baseline data for effective water quality monitoring and mitigation in a region that has the potential to be a health risk hotspot. Some of the specific conclusions are as follows:

(i) The groundwater samples (Ca-Mg-HCO_3_ facies) reflected interactions with limestone and Ca-bearing volcanic lithologies. Both the δ^18^O and δ^2^H variations were similar to the global and local precipitations. Fluoride (2.5–9.9 mg/L) above the WHO recommended limit grouped 20% samples as possible promotor of dental fluorosis and 80% samples as promotor of both dental and skeletal fluorosis. Nitrate of 10–50 mg/L in 25% and > 50 mg/L in 10% samples indicated anthropogenic impact possibly from synthetic fertilizers and sewage leakage. Nitrate Pollution Index (NPI) categorized 65% of the groundwater samples as unpolluted or clean and DWQI values categorized 55% samples as good for drinking even with F^−^ above 1.5 mg/L.
(ii) Lake water (Na-Cl facies) in its concentrated state was unsuitable for drinking and it suggested dominant influence of evaporation. Evaporation also led to depleted d-excess (− 40.80 to − 38.20‰), and enriched δ^18^O (10.98–11.08‰) and δ^2^H (47.4–50.4‰) compared to the global and local precipitations. Fluoride (12.7–13.3 mg/L) contents were similar to the water resources (F^−^ > 10 mg/L) promoting crippling skeletal fluorosis. Lack of nitrate indicated the loss of N through ammonia volatilization in alkaline conditions.
(iii) Estimations of non-carcinogenic risks through HQ_fluoride_ suggested potential non-carcinogenic concerns in order of older adults > elderly pregnant women > children > infants from F^−^ enrichment in the groundwater. The alkaline environment facilitated its mobility from fluoride-bearing lithologies and the evaporation enriched it further in the surface water body. The fluoride-bearing minerals need further research in terms of mineralogical and geochemical studies of the limestone and volcanic deposits. HQ_nitrate_ in groundwater suggested non-carcinogenic concerns for older adults and elderly pregnant women from only 10% samples, and these wells need immediate attention through regulation of fertilizers in the surrounding agricultural fields and minimizing of sewage discharge into the aquifer. None, however, exposed the infants to possible methemoglobinemia. Water from the wells with nitrate between 10–50 mg/L and above 50 m/L might would require disinfection techniques before its consumption.

## Supplementary Information

Below is the link to the electronic supplementary material.

Supplementary file1 (XLSX 13 KB)

## Data Availability

Data used in this study are included in the supplementary file.

## References

[CR1] Abascal, E., Gómez-Coma, L., Ortiz, I., & Ortiz, A. (2022). Global diagnosis of nitrate pollution in groundwater and review of removal technologies. *Science of the Total Environment,**810*, 152233. 10.1016/j.scitotenv.2021.152233 34896495 10.1016/j.scitotenv.2021.152233

[CR2] Adimalla, N., Li, P., & Qian, H. (2019a). Evaluation of groundwater contamination for fluoride and nitrate in semi-arid region of Nirmal Province, South India: A special emphasis on human health risk assessment (HHRA). *Human and Ecological Risk Assessment: An International Journal,**25*(5), 1107–1124. 10.1080/10807039.2018.1460579

[CR3] Adimalla, N., Venkatayogi, S., & Das, S. V. G. (2019b). Assessment of fluoride contamination and distribution: A case study from a rural part of Andhra Pradesh, India. *Applied Water Science,**9*, 1–15. 10.1007/s13201-019-0968-y

[CR4] Ahsan, A., Ahmed, T., Uddin, M. A., Al-Sulttani, A. O., Shafiquzzaman, M., Islam, M. R., & Masria, A. (2023). Evaluation of water quality index (WQI) in and around Dhaka city using groundwater quality parameters. *Water,**15*(14), 2666. 10.3390/w15142666

[CR5] Alarcón-Herrera, M. T., Martin-Alarcon, D. A., Gutiérrez, M., Reynoso-Cuevas, L., Martín-Domínguez, A., Olmos-Márquez, M. A., & Bundschuh, J. (2020). Co-occurrence, possible origin, and health-risk assessment of arsenic and fluoride in drinking water sources in Mexico: Geographical data visualization. *Science of the Total Environment,**698*, 134168. 10.1016/j.scitotenv.2019.134168 31505353 10.1016/j.scitotenv.2019.134168

[CR6] Aragaw, T. T., & Gnanachandrasamy, G. (2021). Evaluation of groundwater quality for drinking and irrigation purposes using GIS-based water quality index in urban area of Abaya-Chemo sub-basin of Great Rift Valley, Ethiopia. *Applied Water Science,**11*(9), 148. 10.1007/s13201-021-01482-6

[CR7] Alvarado, J., Siqueiros-García, J. M., Ramos-Fernández, G., García-Meneses, P. M., & Mazari-Hiriart, M. (2022). Barriers and bridges on water management in rural Mexico: From water-quality monitoring to water management at the community level. *Environmental Monitoring and Assessment,**194*(12), 912. 10.1007/s10661-022-10616-5 36253623 10.1007/s10661-022-10616-5 PMC9579668

[CR8] Alvarez, M. P., & Carol, E. (2019). Geochemical occurrence of arsenic, vanadium and fluoride in groundwater of Patagonia, Argentina: Sources and mobilization processes. *Journal of South American Earth Sciences,**89*, 1–9. 10.1016/j.jsames.2018.10.006

[CR9] ATSDR (Agency for Toxic Substances and Disease Registry) (2023). Exposure Dose Guidance for Water Ingestion. Atlanta, GA: U.S. Department of Health and Human Services, Public Health Service, Jan 31

[CR10] Badeenezhad, A., Radfard, M., Abbasi, F., Jurado, A., Bozorginia, M., Jalili, M., & Soleimani, H. (2021). Effect of land use changes on non-carcinogenic health risks due to nitrate exposure to drinking groundwater. *Environmental Science and Pollution Research,**28*, 41937–41947. 10.1007/s11356-021-13753-5 33797047 10.1007/s11356-021-13753-5

[CR11] Badeenezhad, A., Tabatabaee, H. R., Nikbakht, H. A., Radfard, M., Abbasnia, A., Baghapour, M. A., & Alhamd, M. (2020). Estimation of the groundwater quality index and investigation of the affecting factors their changes in Shiraz drinking groundwater, Iran. *Groundwater for Sustainable Development,**11*, 100435. 10.1016/j.gsd.2020.100435

[CR12] Betancourt-Lineares, A., Irigoyen-Camacho, M. E., Mejía-González, A., Zepeda-Zepeda, M., & Sánchez-Pérez, L. (2013). Prevalencia de fluorosis dental en localidades mexicanas ubicadas en 27 estados y el D.F. a seis años de la publicación de la Norma Oficial Mexicana para la fluoruración de la sal. *Revista De Investigación Clínica,**65*, 237–247. 23877811

[CR13] Calleros, E. Y., Alarcón, M. T., Pérez, R., Cueto, J. A., Moran, J., & Sanín, L. H. (2012). Evaluación de riesgo sistémico y niveles de metahemoglobina en niños que consumen agua contaminada por nitratos. *Ingeniería,**16*(3), 183–194.

[CR14] Can-Chulim, Á., Ortega-Escobar, H. M., García-Calderón, N. E., Reyes-Ortigoza, A. L., González-Hernández, V., & Flores-Román, D. (2011). Origen y calidad del agua subterránea en la cuenca oriental de México. *Terra Latinoamericana,**29*(2), 189–200.

[CR15] Clark, I. D., & Fritz, P. (1997). *Environmental isotopes in hydrogeology*. CRC Press/Lewis Publishers.

[CR16] Chung, S. Y., Rajesh, R., Venkatramanan, S., Selvam, S., Ranganathan, P. C., Oh, Y. Y., & Hussam, E. E. (2020). Processes and characteristics of hydrogeochemical variations between unconfined and confined aquifer systems: A case study of the Nakdong River Basin in Busan City. *Korea. Environ Sci Pollut Res.,**27*, 10087–10102. 10.1007/s11356-019-07451-6 10.1007/s11356-019-07451-6 31933072

[CR17] CONAGUA (2022). Estadísticas del Agua en México 2021. Comisión Nacional del Agua, Ciudad de México (In spanish). https://sinav30.conagua.gob.mx:8080/PDF/EAM_2021.pdf

[CR18] CONAGUA (2023). Actualización de la disponibilidad media anual de agua en el acuífero Libres-Oriental (2102), Estado de Puebla. Subdirección General Técnica Genercia de Aguas Subterráneas. Comisión Nacional del Agua, Ciudad de México (In spanish).

[CR19] Coplen, T. B., Qi, H., Tarbox, L., Lorenz, J., & Buck, B. (2014). USGS 46 Greenland Ice Core Water–A New Isotopic Reference Material for δ2H and δ18O Measurements of Water. *Geostandards and Geoanalytical Research,**38*(2), 153–157. 10.1111/j.1751-908X.2013.00267.x

[CR20] Craig, H. (1961). Isotopic variations in meteoric waters. *Science,**133*, 1702–1703. 17814749 10.1126/science.133.3465.1702

[CR21] Crundwell, F. K. (2017). On the mechanism of the dissolution of quartz and silica in aqueous solutions. *ACS Omega,**2*(3), 1116–1127. 10.1021/acsomega.7b00019 31457494 10.1021/acsomega.7b00019 PMC6641193

[CR22] Cui, J., Tian, L., Biggs, T. W., & Wen, R. (2017). Deuterium-excess determination of evaporation to inflow ratios of an alpine lake: Implications for water balance and modeling. *Hydrological Processes,**31*(5), 1034–1046. 10.1002/hyp.11085

[CR23] Das, R., Rao, N. S., Sahoo, H. K., & Sakram, G. (2023). Nitrate contamination in groundwater and its health implications in a semi-urban region of Titrol block, Jagatsinghpur district, Odisha, India. *Physics and Chemistry of the Earth, Parts a/b/c,**132*, 103424. 10.1016/j.pce.2023.103424

[CR24] de León-Gómez, H., Martin del Campo-Delgado, M. A., Esteller-Alberich, M. V., Velasco-Tapia, F., Alva-Niño, E., & Cruz-López, A. (2020). Assessment of nitrate and heavy metal contamination of groundwater using the heavy metal pollution index: Case study of Linares, Mexico. *Environmental Earth Sciences,**79*, 1–19. 10.1007/s12665-020-09164-3

[CR25] de Oca, R. M. G. F. M., Ramos-Leal, J. A., Solache-Ríos, M. J., Martínez-Miranda, V., & Fuentes-Rivas, R. M. (2019). Modification of the relative abundance of constituents dissolved in drinking water caused by organic pollution: A case of the Toluca Valley, Mexico. *Water, Air, & Soil Pollution,**230*, 1–13. 10.1007/s11270-019-4210-1

[CR26] Dey, R. K., Swain, S. K., Mishra, S., Sharma, P., Patnaik, T., Singh, V. K., & Patel, R. K. (2012). Hydrogeochemical processes controlling the high fluoride concentration in groundwater: A case study at the Boden block area, Orissa, India. *Environmental Monitoring and Assessment,**184*, 3279–3291. 10.1007/s10661-011-2188-2 21713470 10.1007/s10661-011-2188-2

[CR27] Dissanayake, C. B. (1991). The fluoride problem in the ground water of Sri Lanka—environmental management and health. *International Journal of Environmental Studies,**38*(2–3), 137–155. 10.1080/00207239108710658

[CR28] ENCD (2018). Informe Ejecutivo 2011–2014. Encuesta Nacional de Caries y Fluorosis Dental, Centro Nacional de Programas de Preventivos y Control de Enfermidades. Secrretaria de Salud (In Spanish). https://www.gob.mx/cms/uploads/attachment/file/422450/Informe_de_Caries_Dental__Encuesta_Nacional_de_Caries_y_Fluorosis_Dental_2011-2014_1.pdf

[CR29] Esteller, M. V., Kondratenko, N., Expósito, J. L., Medina, M., & Del Campo, M. M. (2017). Hydrogeochemical characteristics of a volcanic-sedimentary aquifer with special emphasis on Fe and Mn content: A case study in Mexico. *Journal of Geochemical Exploration,**180*, 113–126. 10.1016/j.gexplo.2017.06.002

[CR30] Eugster, H. P., & Hardie, L. A. (1978). Saline lakes. In A. Lerman (Ed.), *Lakes: Chemistry, geology, physics (pp 237–293)* (pp. 237–294). Springer-Verlag.

[CR31] Fayiga, A. O., & Nwoke, O. C. (2016). Phosphate rock: Origin, importance, environmental impacts, and future roles. *Environmental Reviews,**24*(4), 403–415. 10.1139/er-2016-0003

[CR32] Fewtrell, L. (2004). Drinking-water nitrate, methemoglobinemia, and global burden of disease: A discussion. *Environmental Health Perspectives,**112*, 1371–1374. 10.1289/ehp.7216 15471727 10.1289/ehp.7216 PMC1247562

[CR33] Gibbs, R. J. (1970). Mechanism controlling world water chemistry. *Science,**170*, 1088–1090. 17777828 10.1126/science.170.3962.1088

[CR34] Gradilla-Hernández, M. S., de Anda, J., Garcia-Gonzalez, A., Meza-Rodríguez, D., Yebra Montes, C., & Perfecto-Avalos, Y. (2020). Multivariate water quality analysis of Lake Cajititlán. *Mexico. Environmental Monitoring and Assessment,**192*(1), 5. 10.1007/s10661-019-7972-4 10.1007/s10661-019-7972-4 31797222

[CR35] Gupta, S. K., Gupta, R. C., Gupta, A. B., Seth, A. K., Bassin, J. K., & Gupta, A. (2000). Recurrent acute respiratory tract infections in areas with high nitrate concentrations in drinking water. *Environmental Health Perspectives,**108*, 363–366. 10.1289/ehp.00108363 10753096 10.1289/ehp.00108363 PMC1638033

[CR36] Hamma, B., Alodah, A., Bouaicha, F., Bekkouche, M. F., Barkat, A., & Hussein, E. E. (2024). Hydrochemical assessment of groundwater using multivariate statistical methods and water quality indices (WQIs). *Applied Water Science,**14*(2), 33. 10.1007/s13201-023-02084-0

[CR37] He, X., Wu, J., & He, S. (2019). Hydrochemical characteristics and quality evaluation of groundwater in terms of health risks in Luohe aquifer in Wuqi County of the Chinese Loess Plateau, northwest China. *Human and Ecological Risk Assessment: An International Journal,**25*, 32–51. 10.1080/10807039.2018.1531693

[CR38] Huang, G., Liu, C., Zhang, Y., & Chen, Z. (2020). Groundwater is important for the geochemical cycling of phosphorus in rapidly urbanized areas: A case study in the Pearl River Delta. *Environmental Pollution,**260*, 114079. 10.1016/j.envpol.2020.114079 32014754 10.1016/j.envpol.2020.114079

[CR39] Jesuraja, K., Selvam, S., & Murugan, R. (2021). GIS-based assessment of groundwater quality index (DWQI and AWQI) in Tiruchendur Coastal City, Southern Tamil Nadu, India. *Environmental Earth Sciences,**80*, 1–17. 10.1007/s12665-021-09542-5

[CR40] Jia, L., Xin, J., Wu, H., Gong, S., Wu, H., & Zhang, Z. (2023). Enhancing nitrate attenuation in groundwater via selectively applying surface agricultural practices: A novel and sustainable strategy for non-point source pollution mitigation. *Water Research,**239*, 120052. 10.1016/j.watres.2023.120052 37178664 10.1016/j.watres.2023.120052

[CR41] Jung, H., Kim, Y. S., Yoo, J., Han, S. J., & Lee, J. (2023). Identification of nitrate sources in tap water sources across South Korea using multiple stable isotopes: Implications for land use and water management. *Science of the Total Environment,**864*, 161026. 10.1016/j.scitotenv.2022.161026 36549543 10.1016/j.scitotenv.2022.161026

[CR42] Kapembo, M. L., Laffite, A., Bokolo, M. K., Mbanga, A. L., Maya-Vangua, M. M., Otamonga, J. P., & Poté, J. (2016). Evaluation of water quality from suburban shallow wells under tropical conditions according to the seasonal variation, Bumbu, Kinshasa, Democratic Republic of the Congo. *Exposure and Health,**8*, 487–496. 10.1007/s12403-016-0213-y

[CR43] Karunanidhi, D., Aravinthasamy, P., Roy, P., Subramani, T., & Jayasena, H. C. (2024). Nitrate contamination in groundwater and its evaluation of non-carcinogenic health hazards from Arjunanadi River basin, south India. *Groundwater for Sustainable Development,**25*, 101153. 10.1016/j.gsd.2024.101153

[CR44] Karunanidhi, D., Aravinthasamy, P., Roy, P. D., Praveenkumar, R. M., Prasanth, K., Selvapraveen, S., Prasanth, K., Selvapraveen, S., Thowbeekrahman, A., Subramani, T., & Srinivasamoorthy, K. (2020). Evaluation of non-carcinogenic risks due to fluoride and nitrate contaminations in a groundwater of an urban part (Coimbatore region) of south India. *Environmental Monitoring and Assessment,**192*, 1–16. 10.1007/s10661-019-8059-y 10.1007/s10661-019-8059-y 31915929

[CR45] Kimambo, V., Bhattacharya, P., Mtalo, F., Mtamba, J., & Ahmad, A. (2019). Fluoride occurrence in groundwater systems at global scale and status of defluoridation–state of the art. *Groundwater for Sustainable Development,**9*, 100223. 10.1016/j.gsd.2019.100223

[CR46] LaFayette, G. N., Knappett, P. S., Li, Y., Loza-Aguirre, I., & Polizzotto, M. L. (2020). Geogenic sources and chemical controls on fluoride release to groundwater in the Independence Basin, Mexico. *Applied Geochemistry,**123*, 104787. 10.1016/j.apgeochem.2020.104787

[CR47] La Jornada de Oriente (2021). La cuenca Libres–Oriental se ha convertido en un infierno ambiental por granjas e industria. March 15, 2021 (In spanish).https://www.lajornadadeoriente.com.mx/puebla/la-cuenca-libres-oriental-se-ha-convertido-en-un-infierno-ambiental-por-granjas-e-industria/

[CR48] Leng, M. J., Lamb, A. L., Heaton, T. H. E., Marshall, J. D., Wolfe, B. B., Jones, M. D., Holmes, J. A., & Arrowsmith, C. (2006). Isotopes in Lake sediments. In M. J. Leng (Ed.), *Isotopes in palaeoenvironmental research* (pp. 147–184). Springer.

[CR49] Li, Z., Yang, Q., Xie, C., & Lu, X. (2023). Source identification and health risks of nitrate contamination in shallow groundwater: A case study in Subei Lake basin. *Environmental Science and Pollution Research,**30*(5), 13660–13670. 10.1007/s11356-022-23129-y 36136183 10.1007/s11356-022-23129-y

[CR50] Liu, R., Ma, T., Qiu, W., Du, Y., & Liu, Y. (2020). Effects of Fe oxides on organic carbon variation in the evolution of clayey aquitard and environmental significance. *Science of the Total Environment,**701*, 134776. 10.1016/j.scitotenv.2019.134776 31726411 10.1016/j.scitotenv.2019.134776

[CR51] Mahlknecht, J., Horst, A., Hernández-Limón, G., & Aravena, R. (2008). Groundwater geochemistry of the Chihuahua City region in the Rio Conchos Basin (northern Mexico) and implications for water resources management. *Hydrological Processes,**22*(24), 4736–4751. 10.1002/hyp.7084

[CR52] Marandi, A., & Shand, P. (2018). Groundwater chemistry and the Gibbs Diagram. *Applied Geochemistry,**97*, 209–212. 10.1016/j.apgeochem.2018.07.009

[CR53] Marghade, D., Malpe, D. B., & Subba Rao, N. (2015). Identification of controlling processes of groundwater quality in a developing urban area using principal component analysis. *Environmental Earth Sciences,**74*, 5919–5933. 10.1007/s12665-015-4616-z

[CR54] Martínez, J., Ortiz, A., & Ortiz, I. (2017). State-of-the-art and perspectives of the catalytic and electrocatalytic reduction of aqueous nitrates. *Applied Catalysis b: Environmental,**207*, 42–59. 10.1016/j.apcatb.2017.02.016

[CR55] Naik, M. R., Mahanty, B., Sahoo, S. K., Jha, V. N., & Sahoo, N. K. (2022). Assessment of groundwater geochemistry using multivariate water quality index and potential health risk in industrial belt of central Odisha, India. *Environmental Pollution,**303*, 119161. 10.1016/j.envpol.2022.119161 35314207 10.1016/j.envpol.2022.119161

[CR56] Natali, S., Doveri, M., Giannecchini, R., Baneschi, I., & Zanchetta, G. (2022). Is the deuterium excess in precipitation a reliable tracer of moisture sources and water resources fate in the western Mediterranean? New insights from Apuan Alps (Italy). *Journal of Hydrology,**614*, 128497. 10.1016/j.jhydrol.2022.128497

[CR57] Navarro, O., González, J., Júnez-Ferreira, H. E., Bautista, C. F., & Cardona, A. (2017). Correlation of arsenic and fluoride in the groundwater for human consumption in a semiarid region of Mexico. *Procedia Engineering,**186*, 333–340. 10.1016/j.proeng.2017.03.259

[CR58] Ochoa-Rivero, J. M., Jacquez-Herrera, V., Prieto-Amparán, J. A., Loya-Fierro, O., Ballinas-Casarrubias, L., González-Horta, C., & Rocha-Gutiérrez, B. A. (2023). Risk assessment for the distribution and levels of fluoride and nitrate in groundwater in a semi-arid area of northern Mexico. *Groundwater for Sustainable Development,**23*, 101045. 10.1016/j.gsd.2023.101045

[CR59] Panneerselvam, B., Karuppannan, S., & Muniraj, K. (2020). Evaluation of drinking and irrigation suitability of groundwater with special emphasizing the health risk posed by nitrate contamination using nitrate pollution index (NPI) and human health risk assessment (HHRA). *Human and Ecological Risk Assessment: An International Journal,**27*(5), 1324–1348. 10.1080/10807039.2020.1833300

[CR60] Park, Y., Kim, Y., Park, S. K., Shin, W. J., & Lee, K. S. (2018). Water quality impacts of irrigation return flow on stream and groundwater in an intensive agricultural watershed. *Science of the Total Environment,**630*, 859–868. 10.1016/j.scitotenv.2018.02.113 29499541 10.1016/j.scitotenv.2018.02.113

[CR61] Patel, P. S., Pandya, D. M., & Shah, M. (2023). A systematic and comparative study of Water Quality Index (WQI) for groundwater quality analysis and assessment. *Environmental Science and Pollution Research,**30*(19), 54303–54323. 10.1007/s11356-023-25936-3 36940024 10.1007/s11356-023-25936-3

[CR62] Pfahl, S., & Sodemann, H. (2014). What controls deuterium excess in global precipitation? *Climate of the past,**10*(2), 771–781. 10.5194/cp-10-771-2014

[CR63] Piper, A. M. (1944). A graphical procedure in the geochemical interpretation of water analysis. *Transactions American Geophysical Union,**25*, 914–928.

[CR64] Podgorski, J., & Berg, M. (2022). Global analysis and prediction of fluoride in groundwater. *Nature Communications,**13*(1), 4232. 10.1038/s41467-022-31940-x 10.1038/s41467-022-31940-x PMC9343638 35915064

[CR65] Reyes-Gómez, V. M., Gutiérrez, M., Nájera-Haro, B., Núñez-López, D., & Alarcón-Herrera, M. T. (2017). Groundwater quality impacted by land use/land cover change in a semiarid region of Mexico. *Groundwater for Sustainable Development,**5*, 160–167. 10.1016/j.gsd.2017.06.003

[CR66] Rojas Fabro, A. Y., Pacheco Ávila, J. G., Alberich, E., Ma, V., Cabrera Sansores, S. A., & Camargo-Valero, M. A. (2015). Spatial distribution of nitrate health risk associated with groundwater use as drinking water in Merida, Mexico. *Applied Geography,**65*, 49–57. 10.1016/j.apgeog.2015.10.004

[CR67] Roy, P. D., García-Arriola, O. A., & Selvam, S. (2024). Seasonality of hydrogeochemical evolutions and isotopic variabilities (δ18O, δ2H and d-excess) in the surface water as well as groundwater from tropical central-south Mexico. *Environmental Research*. 10.1016/j.envres.2024.118529 10.1016/j.envres.2024.118529 38395335

[CR68] Roy, P. D., García-Arriola, O. A., Selvam, S., Vargas-Martínez, I. G., & Sánchez-Zavala, J. L. (2023). Evaluation of water from Lake Coatetelco in central-south Mexico and surrounding groundwater wells for drinking and irrigation, and the possible health risks. *Environmental Science and Pollution Research,**30*(54), 115430–115447. 10.1007/s11356-023-30488-7 37884711 10.1007/s11356-023-30488-7 PMC10682244

[CR69] Roy, P. D., Selvam, S., Gopinath, S., Logesh, N., Sánchez-Zavala, J. L., & Lakshumanan, C. (2022). Geochemical evolution and seasonality of groundwater recharge at water-scarce southeast margin of the Chihuahuan Desert in Mexico. *Environmental Research,**203*, 111847. 10.1016/j.envres.2021.111847 34384751 10.1016/j.envres.2021.111847

[CR70] Roy, P. D., Selvam, S., Gopinath, S., Lakshumanan, C., Muthusankar, G., Quiroz-Jiménez, J. D., & Venkatramanan, S. (2021). Hydro-geochemistry-based appraisal of summer-season groundwater from three different semi-arid basins of northeast Mexico for drinking and irrigation. *Environmental Earth Sciences,**80*(16), 529. 10.1007/s12665-021-09828-8

[CR71] Sanad, H., Mouhir, L., Zouahri, A., Moussadek, R., El Azhari, H., Yachou, H., Ghanimi, A., Oueld Lhaj, M., & Dakak, H. (2024). Assessment of Groundwater Quality Using the Pollution Index of Groundwater (PIG), Nitrate Pollution Index (NPI), Water Quality Index (WQI), Multivariate Statistical Analysis (MSA), and GIS Approaches: A Case Study of the Mnasra Region, Gharb Plain. *Morocco. Water,**16*(9), 1263. 10.3390/w16091263

[CR72] Sánchez-Gutiérrez, R., Sánchez-Murillo, R., Esquivel-Hernández, G., Birkel, C., Boll, J., Rojas-Jiménez, L. D., & Castro-Chacón, L. (2023). Nitrate legacy in a tropical and complex fractured volcanic aquifer system. *Journal of Geophysical Research: Biogeosciences,**128*(8), e2023JG007554. 10.1029/2023JG007554

[CR73] Sánchez-Murillo, R., González-Hita, L., Mejía-González, M. A., Carteño-Martinez, B., Aparicio-González, J. C., Mañón-Flores, D., & Gimeno, L. (2023). Tracing isotope precipitation patterns across Mexico. *PLOS Water,**2*(10), e0000136. 10.1371/journal.pwat.0000136

[CR74] Servicio Geologico Mexicano (2011a). Carta geológico-minera Guadalupe Victoria. E14-B35, Tlaxcala, Puebla y Veracruz.Scale 1: 250 000 (In spanish).

[CR75] Servicio Geologico Mexicano (2011b). Carta geológico-minera Xonacatlán. E14-B25, Puebla y Veracruz. Scale 1: 250 000 (In spanish).

[CR76] Servicio Geologico Mexicano (2012). Carta geológico-minera Mexcaltepec. E14-B24, Puebla y Tlaxcala. Scale 1: 250 000 (In spanish).

[CR77] Shaji, E., Sarath, K. V., Santosh, M., Krishnaprasad, P. K., Arya, B. K., & Babu, M. S. (2024). Fluoride contamination in groundwater: A global review of the status, processes, challenges, and remedial measures. *Geoscience Frontiers,**15*(2), 101734. 10.1016/j.gsf.2023.101734

[CR78] Subba Rao, N., Sunitha, B., Sun, L., Spandana, B. D., & Chaudhary, M. (2020). Mechanisms controlling groundwater chemistry and assessment of potential health risk: A case study from South India. *Geochemistry,**80*(4), 125568. 10.1016/j.chemer.2019.125568

[CR79] Torres-Martínez, J. A., Mora, A., Mahlknecht, J., Daesslé, L. W., Cervantes-Avilés, P. A., & Ledesma-Ruiz, R. (2021). Estimation of nitrate pollution sources and transformations in groundwater of an intensive livestock-agricultural area (Comarca Lagunera), combining major ions, stable isotopes and MixSIAR model. *Environmental Pollution,**269*, 115445. 10.1016/j.envpol.2020.115445 33277063 10.1016/j.envpol.2020.115445

[CR80] Torres-Martínez, J. A., Mora, A., Knappett, P. S., Ornelas-Soto, N., & Mahlknecht, J. (2020). Tracking nitrate and sulfate sources in groundwater of an urbanized valley using a multi-tracer approach combined with a Bayesian isotope mixing model. *Water Research,**182*, 115962. 10.1016/j.watres.2020.115962 32629319 10.1016/j.watres.2020.115962

[CR81] Tiwari, A. K., Nota, N., Marchionatti, F., & De Maio, M. (2017). Groundwater-level risk assessment by using statistical and geographic information system (GIS) techniques: A case study in the Aosta Valley region, Italy. *Geomatics, Natural Hazards and Risk,**8*, 1396–1406. 10.1080/19475705.2017.1337655

[CR82] Tokazhanov, G., Ramazanova, E., Hamid, S., Bae, S., & Lee, W. (2020). Advances in the catalytic reduction of nitrate by metallic catalysts for high efficiency and N2 selectivity: A review. *Chemical Engineering Journal,**384*, 123252. 10.1016/j.cej.2019.123252

[CR83] United Nations. (2023). Water and Sanitation. Department of Economic and Social Affairs, Sustaiabale Development. https://sdgs.un.org/topics/water-and-sanitation#description

[CR84] USEPA. (2004). Risk Assessment Guidance for Superfund Volume I: Human Health Evaluation Manual (Part E). http://www.epa.gov/oswer/riskassessment/ragse/pdf/introduction.pdf.

[CR85] Vasanthavigar, M., Srinivasamoorthy, K., Rajiv Ganthi, R., Vijayaraghavan, K., & Sarma, V. S. (2010). Characterisation and quality assessment of groundwater with a special emphasis on irrigation utility: Thirumanimuttar sub-basin, Tamil Nadu, India. *Arabian Journal of Geosciences,**5*, 245–258. 10.1007/s12517-010-0190-6

[CR86] Verma, A., Yadav, B. K., & Singh, N. B. (2020). Hydrochemical monitoring of groundwater quality for drinking and irrigation use in Rapti Basin. *SN Applied Sciences,**2*, 1–15. 10.1007/s42452-020-2267-5

[CR87] Wassenaar, L. I., Van Wilgenburg, S. L., Larson, K., & Hobson, K. A. (2009). A groundwater isoscape (δD, δ18O) for Mexico. *Journal of Geochemical Exploration,**102*, 123–136. 10.1016/j.gexplo.2009.01.001

[CR88] WHO (2017). Guidelines for Drinking Water Quality: Fourth Edition Incorporating the First Addendum. World Health Organization, Geneva. https://www.who.int/publications/i/item/9789241549950 28759192

[CR89] Xia, Z., & Winnick, M. J. (2021). The competing effects of terrestrial evapotranspiration and raindrop re-evaporation on the deuterium excess of continental precipitation. *Earth and Planetary Science Letters,**572*, 117120. 10.1016/j.epsl.2021.117120

[CR90] Xiao, Y., Hao, Q., Zhang, Y., Zhu, Y., Yin, S., Qin, L., & Li, X. (2022). Investigating sources, driving forces and potential health risks of nitrate and fluoride in groundwater of a typical alluvial fan plain. *Science of the Total Environment,**802*, 149909. 10.1016/j.scitotenv.2021.149909 34525690 10.1016/j.scitotenv.2021.149909

[CR91] Zamora-Martínez, O., Montaño-Hilario, J. M., Galindo-Zavala, V. B., Siebe-Grabach, C., & Prado-Pano, B. L. (2016). Determinación simultánea de cationes mayoritarios en muestras de agua residual por medio de cromatografía de iones con detección conductimétrica. *Revista Internacional De Contaminación Ambiental,**32*(3), 293–301. 10.20937/RICA.2016.32.03.04

[CR92] Zhou, J., Du, Y., Deng, Y., Tao, Y., Leng, Z., Ma, T., & Wang, Y. (2022). Source identification of groundwater phosphorus under different geological settings in the central Yangtze River basin. *Journal of Hydrology,**612*, 128169. 10.1016/j.jhydrol.2022.128169

